# Updated single cell reference atlas for the starlet anemone *Nematostella vectensis*

**DOI:** 10.1186/s12983-024-00529-z

**Published:** 2024-03-18

**Authors:** Alison G. Cole, Julia Steger, Julia Hagauer, Andreas Denner, Patricio Ferrer Murguia, Paul Knabl, Sanjay Narayanaswamy, Brittney Wick, Juan D. Montenegro, Ulrich Technau

**Affiliations:** 1 Department of Neurosciences and Developmental Biology, Faculty of Life Sciences, University of Vienna, Djerassiplatz 1, 1030 Vienna, Austria; 2 Research Platform Single Cell Regulation of Stem Cells, University of Vienna, Djerassiplatz 1, 1030 Vienna, Austria; 3 UCSC Cellbrowser, University of California, Santa Cruz, USA; 4 Max Perutz Labs, University of Vienna, Dr. Bohrgasse 9, 1090 Vienna, Austria

**Keywords:** Cnidarian development, Neuronal inventory, Transcriptomics, scRNAseq

## Abstract

**Background:**

The recent combination of genomics and single cell transcriptomics has allowed to assess a variety of non-conventional model organisms in much more depth. Single cell transcriptomes can uncover hidden cellular complexity and cell lineage relationships within organisms. The recent developmental cell atlases of the sea anemone *Nematostella vectensis*, a representative of the basally branching Cnidaria, has provided new insights into the development of all cell types (Steger et al Cell Rep 40(12):111370, 2022; Sebé-Pedrós et al. Cell 173(6):1520–1534.e20). However, the mapping of the single cell reads still suffers from relatively poor gene annotations and a draft genome consisting of many scaffolds.

**Results:**

Here we present a new wildtype resource of the developmental single cell atlas, by re-mapping of sequence data first published in Steger et al. (2022) and Cole et al. (Nat Commun 14(1):1747, 2023), to the new chromosome-level genome assembly and corresponding gene models in Zimmermann et al. (Nat Commun 14, 8270 (2023). 10.1038/s41467-023-44080-7). We expand the pre-existing dataset through the incorporation of additional sequence data derived from the capture and sequencing of cell suspensions from four additional samples: 24 h gastrula, 2d planula, an inter-parietal region of the bodywall from a young unsexed animal, and another adult mesentery from a mature male animal.

**Conclusion:**

Our analyses of the full cell-state inventory provide transcriptomic signatures for 127 distinct cell states, of which 47 correspond to neuroglandular subtypes. We also identify two distinct putatively immune-related transcriptomic profiles that segregate between the inner and outer cell layers. Furthermore, the new gene annotation Nv2 has markedly improved the mapping on the single cell transcriptome data and will therefore be of great value for the community and anyone using the dataset.

**Supplementary Information:**

The online version contains supplementary material available at 10.1186/s12983-024-00529-z.

## Introduction

The establishment of single cell transcriptomics in a wide range of organisms that are usually not accessible to genetic manipulation has opened the avenue to make large-scale comparisons of cell type complexity and evolutionary origin of specific cell types. However, a key aspect in such comparative approaches is the quality and depth of the underlying datasets. This largely depends on the quality of the genome and the corresponding gene annotations. For the sea anemone *Nematostella vectensis*, which is now one of the major model organisms among cnidarians, single cell transcriptomes have been published, which cover the developmental stages from the gastrula to the adult stage [1–3]. However, the underlying single cell reads have been mapped on a draft genome, consisting of numerous scaffolds and a reference transcriptome, called Nve, which does not always include the 3’ UTR required for mapping of reads derived from the 10X Genomics platform. As a result, while we artificially extended the gene models in the 3prime direction, some genes may have been erroneously annotated. Our group recently generated a new chromosome-scale assembly and—using PacBio Isoseq reads—an improved, new gene annotation, called Nv2 [4]. The new gene annotation requires a new mapping tool to fully explore the single cell dataset.

Here we present a new wildtype single cell transcriptome resource for the anthozoan model *Nematostella vectensis*, based on the re-mapping of sequence data first published in Steger, Denner, Cole, et al. 2022 [1] and Cole, Jahnel et al., 2023 [3], to the new chromosome-level genome assembly and corresponding gene models in [4]. We further expand the pre-existing dataset through the incorporation of additional sequence data derived from the capture and sequencing of cell suspensions from three additional samples: 24 h gastrula, 2d planula, and another adult mesentery from a mature male animal. In general, we recapitulate the previously reported features of the dataset, and expand our cluster annotations from the previous publications. We provide an in-depth analysis of the three main tissue layers: the inner gastrodermis, the outer epithelium, and the cells from the boundary between these that contribute to the pharynx and septal filaments. We make the revised analyzed dataset available for public exploration on the UCSC single cell web browser (sea-anemone-atlas.cells.ucsc.edu).

## Results and discussion

We analyzed the re-mapped data in two phases: (1) we merged all samples together to generate the complete dataset and clustered the full dataset into tissue-level partitions; (2) we analyzed each data partition separately to arrive at a complete cell-state atlas. We then copy these cell-state identifiers into the merged dataset for further use in data exploration (Additional file 2: Supplementary Data1: sample.metadata), and we provide here expression data plots separated between the developmental data derived from 18 h post-fertilization to 16day primary polyps: (“developmental”), and the adult tissue samples (“adult tissues”). Our results do not differ greatly from the cell type annotations provided in [1]. Rather we provide here a finer-grain description of the cell-state inventory present across the life history stages.

**Fig. 1:**
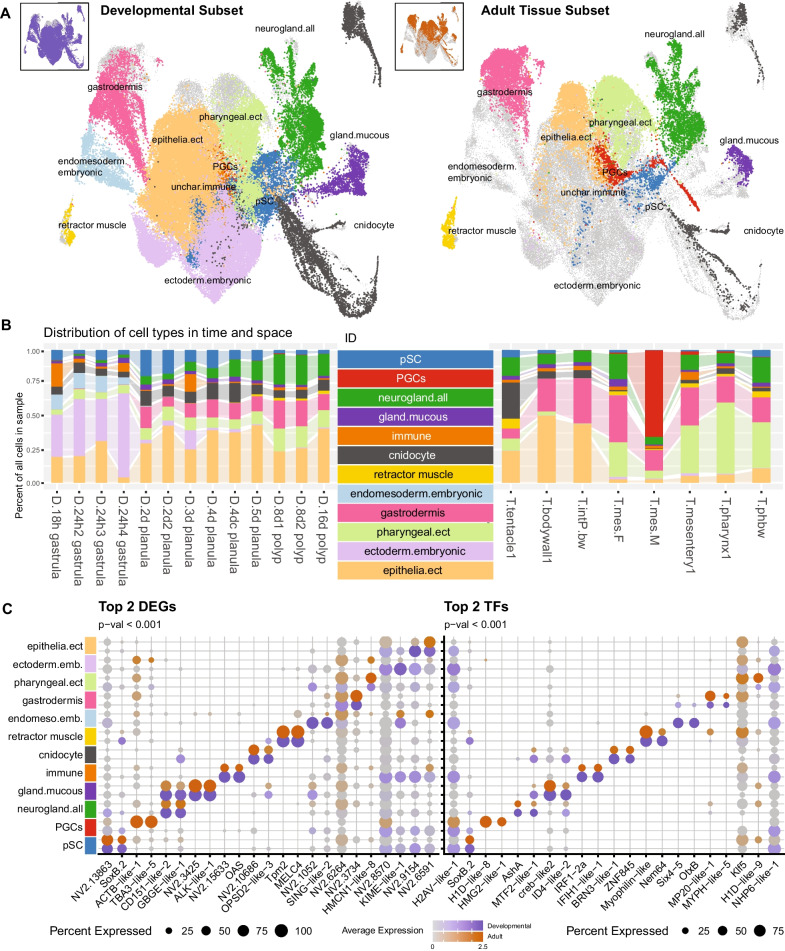
Updated cell atlas for the starlet sea anemone *Nematostella vectensis*. **A** Dimensional reduction cell map (UMAP) illustrating clustering of cells from the developmental (left) or adult tissue (right) subsets. Color of clustering corresponds to the legend in (b). Distribution of the entire subset is shown in the inset. **B** Relative contribution of each library to the clusters from the developmental (left) or tissue (right) samples. **C**) Dotplot representation of differentially expressed gene sets from all genes (left) and restricted to transcription factors only (right). Expression profiles are split between the two subsets. Orange scale = Adult subset; Dark slate blue scale = Developmental subset

We recapitulated our previous analysis and recover the same principal epithelial partitions and all neuroglandular derivatives present (Fig. 1). Both the inner gastrodermis and the outer epithelial layer show clear developmental maturation as evidenced by the clustering of two distinct embryonic cell partitions within the dataset (Fig. 1A, B: ‘endomesoderm.embryonic’ and ‘ectoderm.embryonic’). This is not likely to be an unresolved batch effect, as we find cells of the mature state contributed from all libraries, while a distinct early profile is associated with cells predominantly from the gastrula stages (see sample distributions in Additional file 1: Figs. S1, S2, S4, S5).

### Epithelia

**Fig. 2:**
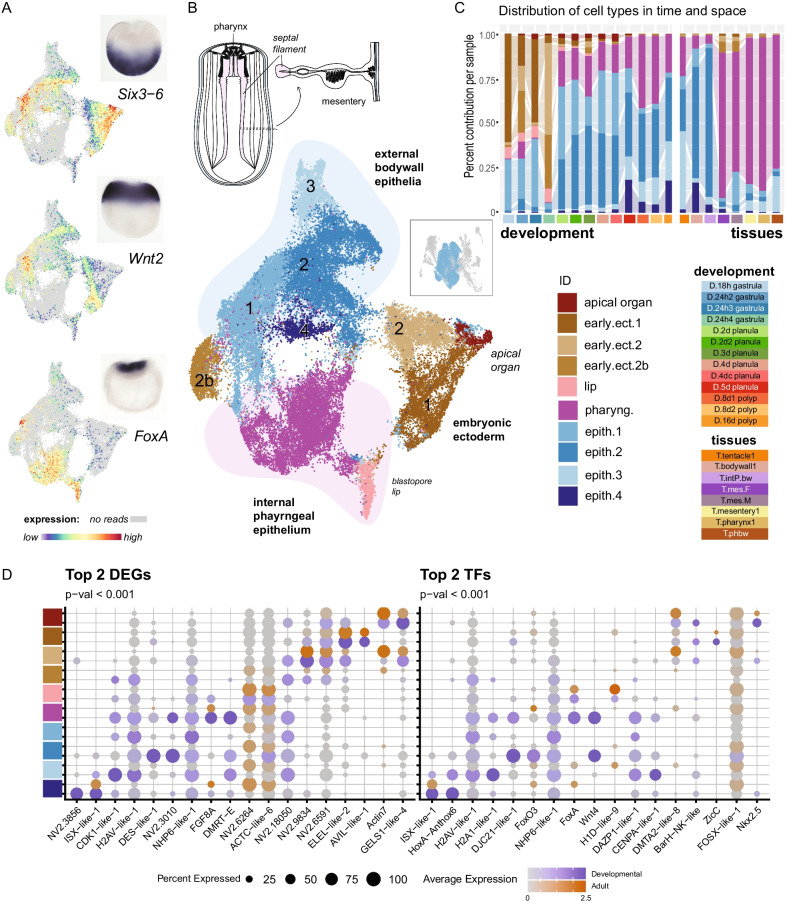
Early embryonic ectoderm matures into two distinct tissues. **A** Sorting according to ectodermal patterning within the transcriptomic data is evidenced by comparing expression patterns known from in situ hybridization with expression in the dimensional reduction. **B** Schematic illustrates the locations of the ectodermal layers in the adult. UMAP dimensional reduction of the ectodermal partition colored by cell state identity highlighting the retention of both spatial and temporal organisation within the clustering. **C** Relative distribution of cell cluster identities across all samples in the dataset. **E** Dotplot representation of differentially expressed gene sets from all genes (left) and restricted to transcription factors only (right). Expression profiles are split between the two subsets. Orange scale = Adult subset; Dark slate blue scale = Developmental subset

**Fig. 3:**
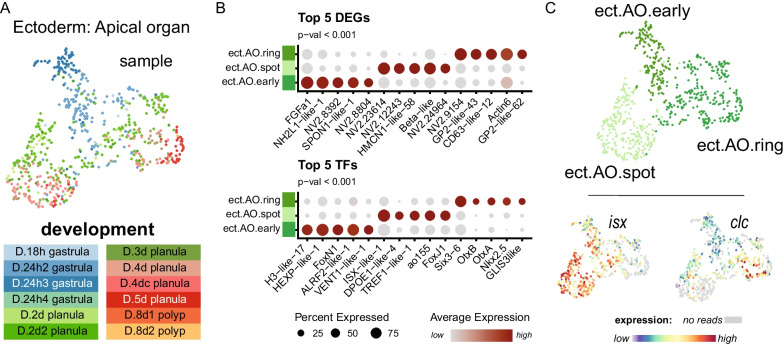
Cell of the apical organ of planula are identifiable from transcriptomic profiles. **A** Sample distribution of apical organ cells shown on UMAP reduction **B** Dotplot of differentially expressed genes (top) and transcription factors (bottom) **C** Cell state identities of apical organ cells (top) and known regional markers (bottom)

The largest partition in our dataset is the ectodermal epithelial cells. The broad underlying groups of ectodermal cells include the embryonic ectoderm, the external body wall epithelium, and the internal pharyngeal ectoderm (Fig. 1A). The ectodermal oral-aboral body axis is established by a gradient of Wnt/ß-catenin signaling during early embryonic development, which leads to the specification of three major expression domains: the oral domain, a midbody domain and the aboral domain [5–7]. Notably, although the dissociation is thought to abolish all spatial information, the analysis of our data shows that the spatial information along the primary body axis is retained in defined clusters of our gastrula ectoderm subset, with well-known signalling pathways and patterning genes demarcating the oral (*FoxA,*), central (*Wnt2*) and aboral (*Six3-6)* domains (Fig. 2A) [8–10]. The proportions of these domains are consistent between spatial patterns and our sc-transcriptomic data (Fig. 2A). Furthermore, the embryonic epithelium widely expresses *Ptx1*, a maternally deposited toxin that is important for the protection from predators (Additional file 1: Fig. S1 and Additional file 2: Supplementary Data 1; [11, 12]. Clustering of the ectodermal subset retains separation of the three primary partitions, further emphasizing the changes in transcriptomic profiles as the ectoderm matures (Fig. 2B, C). The embryonic partition includes three cell states that show differential distribution across the gastrula libraries, and one that corresponds to the apical organ in the aboral domain (Fig. 2C, Additional file 1: Fig. S1). The aboral pole of the planula larva is characterized by a small region with a long ciliary tuft commonly referred to as the apical organ (ao). It is a transient structure that disintegrates during metamorphosis, and likely preforms sensory functions [13–15]. These cells first appear in the 24 h gastrula, are sparsely detected in the 5d sample, and are absent again from the 8d primary polyp samples (Figs. 2C, 3A). Early apical tuft cells from the gastrula samples are enriched for *fgf1a*, *FoxN1*, and *vent1-like* expression (*ect.AO.early*: Fig. 2D). We further identify two apical organ-related cell types corresponding to centrally located apical organ cells enriched in *isx* and *foxJ1* expression (spot) and the surrounding sensory neurons (*slc* expression: ring [15]) (Fig. 3B, C, Additional file 2: Supplementary Data 1).

Within the mature epithelial ectodermal partition (blues), we identify four cell states (Fig. 2B). Epithelial cell state 1 and 2 are enriched in the bodywall samples and state 3 is enriched in the tentacle sample (Fig. 2C). Cell state 4 expresses markers of putative neural precursor identity, including *nkx2.5* and *soxB*(2) (Fig. 2D, Additional file 2: Supplementary Data 1). We can further differentiate 7 cell states in the isolated partition analysis, 4 of which show distinct transcriptomic properties indicative of ciliated cells (*foxJ1*: yellow), progenitor cells (*myc2*: dark grey), actin-enriched (green: ect.3) and a population expressing mitotic markers (histone-rich) (Additional file 1: Fig. S2, Additional file 2: Supplementary Figures and Data 1). The ectoderm at the boundary with the gastrodermis has unique properties in terms of both its developmental formation, and its contribution to the adult. Cells from this region form the ectodermal portion of the pharynx as well as lining the distal-most portion of the mesentery folds. This territory is enriched in digestive gland cells and has been proposed to be homologous to the bilaterian endoderm [16]. In support of this hypothesis, this territory can be identified throughout development by the expression of the transcription factor *foxA*; the vertebrate *foxa2* ortholog is a marker of the definitive endoderm [17, 18]. Within the *foxA* positive pharyngeal ectoderm and septal filament territory we also further identify 7 cell states, including an additional population expressing mitotic markers (histone-rich), a population of early blastopore lip cells, a DLGP5-positive cell state, and a LRWamide-sensitive (LRWa-receptor expressing) DMBX1-positive cell state (Additional file 1: Fig. S3).

### Gastrodermis

**Fig. 4:**
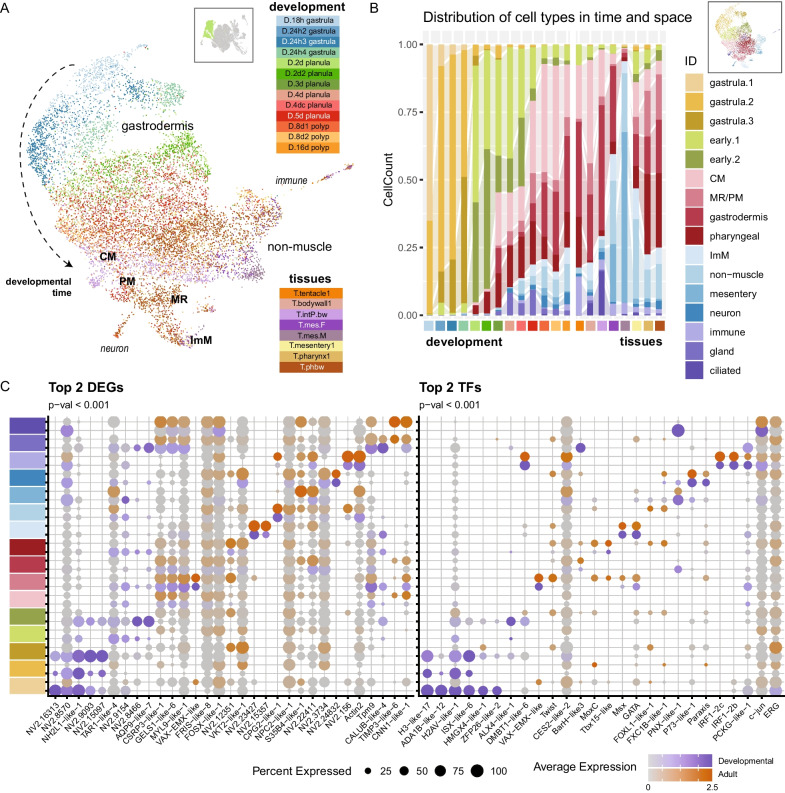
Inner cell layer matures into gastrodermis and derived cell types. **A** UMAP dimensional reduction cell plot, colored by sample origin. Inset: included partitions. Dashed arrow: maturation pathway. CM: circular muscle; PM: parietal muscle; MR: mesentery retractor muscle; ImM: intermuscular membrane. **B** Bar plot representation of fraction of cells from each cluster (colours) within each sample (bars). **C** Dot plot representation of expression profiles of up-regulated genes (left) and up-regulated transcription factors (right) across each cluster. Expression separated between cells of the developmental series (dark slate blue scale) and the adult tissue series (orange scale). Grey indicates average scaled expression of 0 or below. See Supplementary material for full gene lists

**Fig. 5:**
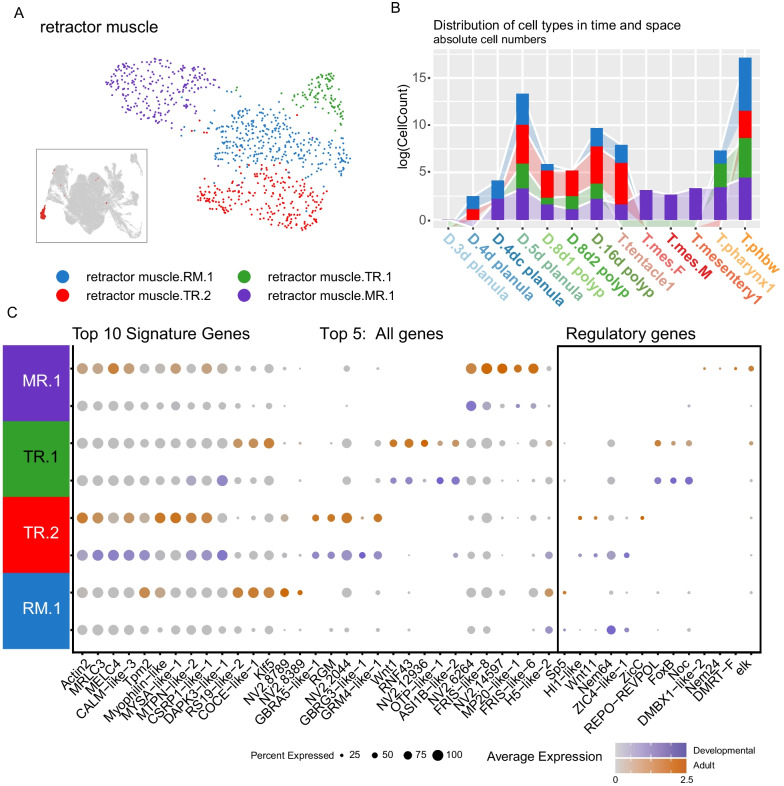
Sub-states of retractor muscle cells from both germ layers can be resolved. **A** Retractor muscle partition within the entire dataset (inset). UMAP representation of four unique cluster states. **B** Bar plot showing the log count (**C**) of cells from each sample contributing to the partition. **C** Dot plot of gene expression separated between partition cluster cells from the developmental (dark slate blue gradient) or adult tissue (orange gradient) samples. Expression profiles of the differentially expressed genes across the partition (Signature Genes) and each partition cluster (Top 5 All Genes), and its regulatory profile (box)

One of the first cell fate decisions in the embryo is the specification of the germ layers, wherein derivatives of the inner cell layer, commonly called (mes)endoderm (but see [16] for an alternative view), are identifiable in their mature state by expression of high levels of specific soma ferritins (FRIS-like) and filamentous collagens presumably in preparation of the forming mesoglea (Additional file 1: Fig. S5 and Additional file 2: Supplementary Data 1). As noted for the ectodermal partition, there is a strong maturation signal evident also in the inner cell layer (Fig. 4A). The early gastrodermis has a unique transcriptomic signature rich in histones genes indicative of active proliferation, and the three identified cell states do not have unique expression profiles (Additional file 1: Fig. S4). There is an intermediate maturation phase at 2d (green clusters, Fig. 4), wherein also the pharyngeal gastrodermis is detectable (dark red, Fig. 4B). This state is then replaced by differentiation of various cell types that are maintained into the adult. The mature gastrodermis itself is characterized by the lack of an additional differentially expressed gene set when compared to the remaining mesendodermal cell (Fig. 4C). The transcriptomic profile corresponding to the mature gastrodermis of the pharynx appears at 2d, the mature gastrodermis profile appears at day 3, while at 4d the non-muscle mesendoderm is first evident, characterized by the expression of the large glycoprotein *cpg2-like-1*, the lectin *csl3-like-3*, as well as phospholipase PA2 orthologs (PA2-like9). As the inner layer matures, differentiated cell types emerge. We detected ciliated cells associated with the body wall samples, secretory cells with a gland-like profile (expressing *mucin*), a putatively neural population (expressing *LRWamide-1*), and a distinct immune state which expresses IRF orthologs (expressing *irf1-2b* and i*rf1-2c*) (Fig. 4C, Additional file 1: Fig. S5 and Additional file 2: Supplementary Data 1). These latter cells also express *snailB*, which transitions to a single-cell expression territory post-gastrulation. This cell state is discussed further below. The mature inner cell layer is rich in apolipophorins, indicative of nutrient storage and mobilization expected of the gastrodermis. Within this layer we can identify one cell state that expresses *twist* and *VAX-EMX-like,* which represents the pharyngeal gastrodermis where these gene have been reported to be expressed [3, 16]. Like that described for the adult tissue-only samples [3], cells corresponding to the circular musculature, the inter-muscular membrane, and the parietal and early mesentery retractors are identifiable within the dataset (Fig. 4A). At the resolution explored here for clustering, the parietal and early mesentery cells cluster together. Mature mesentery retractor cells are found rather within the retractor muscle cluster itself (Fig. 5).

### Retractor muscle

The retractor muscle profile can be separated into four distinct profiles, two of which are indicative of earlier (TR.1: *rnf43*) and later states (TR.2: *gbra5*; *grbg*3) of the tentacle retractor muscle (*nem64*), one of the mesentery retractor (MR.1: *nem24*), and one stable mature cell state that is a convergent profile of both fast muscles types as has been previously described [3] (Fig. 5). While the mature profile is shared across all clusters, the ectodermally-derived tentacle muscle expresses GABA/Glutamate receptors that are absent from the mesentery counterpart. We previously demonstrated that the ectodermal tentacle muscle likely derives from the neuroglandular progenitor (NPC) lineage, as the tentacle retractor cells express *soxB*(2) (also called *SoxB2a)* [3] and populations of *nem64* expressing cells can be found within the putative stem cell partition (Fig. 7; [1]). The early tentacle profile associated with the differentiated retractor muscle here expresses *wnt1* and is restricted to samples obtained from the pharynx-region of the adult. Interestingly these cells also express *rnf43*, a zinc finger known to negatively regulate wnt signaling in other systems [19, 20]. Previously both FGF [21] and Notch [22] signaling have been demonstrated to play a role in tentacle outgrowth. Wnt signalling is crucial for establishing the oral pole in the embryo and indeed acts as an organizer capable of inducing ectopic head formation, including tentacle outgrowth, in both embryos [5] and regenerating adults [23, 24]. Wnt expression within tentacle-specific cell types, as shown here for the tentacle muscle cells, indicates a role for wnt signaling in regulating tentacle formation in a cell-type specific manner.

### Cnidocytes

**Fig. 6:**
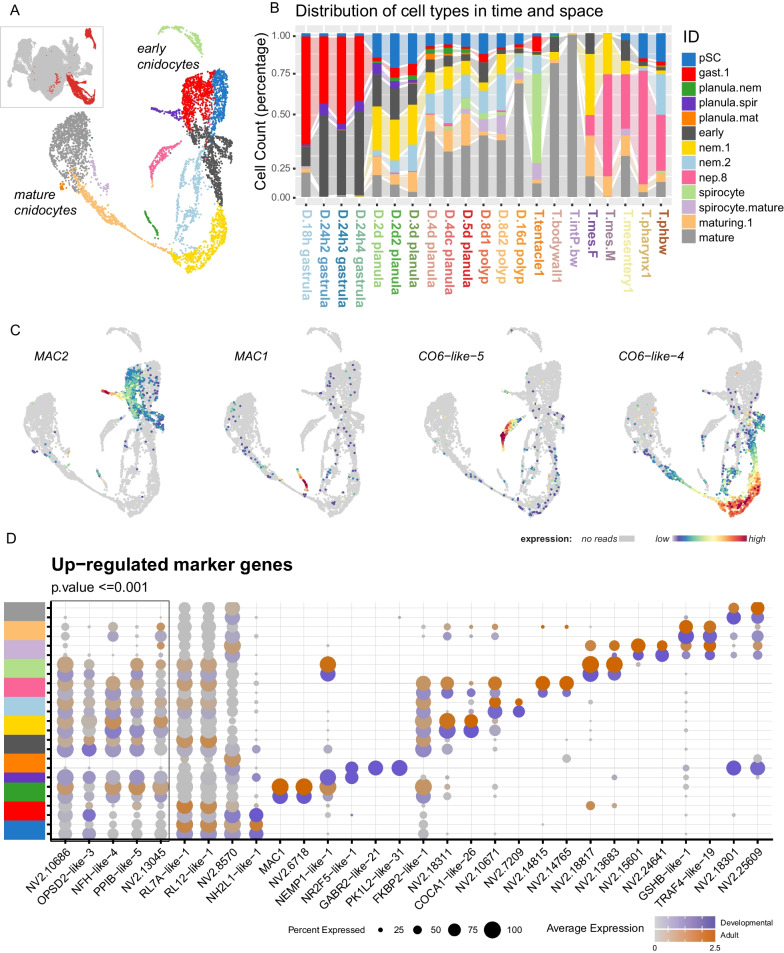
Cnidocyte specification pathways are recovered. **A** Cell plot indicating the clusters of the cnidocyte partition (inset). **B** Distribution of sample contribution to each identified cluster. **C** Specific toxin profiles associated with cnidocyte subtypes. **D** Dotplot of marker expression (box) and differentially up-regulated genes of each cluster. Expression separated between cells of the developmental series (Dark slate blue scale) and the adult tissue series (orange scale). Grey indicates average scaled expression of 0 or below. See Supplementary material for full gene lists

Cnidocyte trajectories described in Steger et al. 2022 [1] are recovered (Fig. 6). Here, a larger portion of the putative stem cells are included in this cluster (cnidocyte.pSC: *sox3* positive; Additional file 2: Supplementary Data 1), reflective of the fuzzy cluster boundaries inherent in these methods. The different cnidocyte sub-types express different members of the membrane attack complex (*mac1* and *mac2*) (Fig. 6C). Updated gene lists representing the expression profiles associated with the distinct phases of the specification trajectories are available here with the updated genome mapping (Additional file 2: Supplementary Data 1).

### pSC and PGCs

**Fig. 7:**
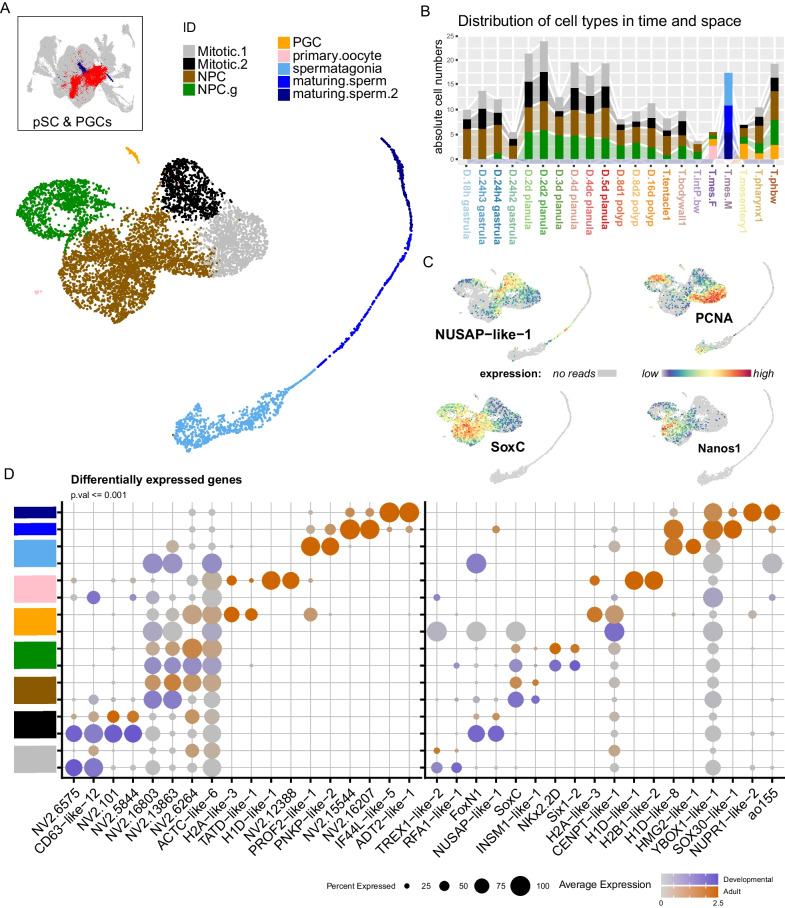
Neuroglandular precursor cells can be identified exiting from the mitotic cycle and derive from both germ layers. **A** Identified clusters. (inset) Partitions included in the analyses: Putative stem cells (red) and primary germ cells (blue) **B** Distribution of cells from each sample included in the cluster: absolute cell counts **C** expression profiles of mitotic markers of DNA-synthesis (*PCNA*) and division (*NUSAP-like-1*), and NPC marker *soxC* and pre-neural marker *nanos1*. **D** Dotplot expression of top marker genes (left) and differentially expressed transcription factors (right) from each cluster. Expression separated between cells of the developmental series (Dark slate blue scale) and the adult tissue series (orange scale). Grey indicates average scaled expression of 0 or below. See Supplementary material for full gene lists

We identify two partitions that contain the putative stem cells (pSC) and the primary germ cells (PGCs; Fig. 7A inset). The latter were previously identified as part of the pSC cluster, and it was suggested that this was the first fate choice facing the stem cell population upon exit from the mitotic cycle [1]. Here we include an additional mature mesentery from a male specimen, thereby enriching for spermatogonia. The germ cell cluster is predominantly composed of maturing sperm from this male mesentery, but also contains early oocytes and stem cells (Additional file 1: Fig. S7) and derive from the pharynx and female mesentery libraries, consistent with what has been described for early PGC formation in this system [25]. This subset contains a full maturation sequence of spermatogenesis and a small cluster of primary oocytes, and early state primary germ cells (PGC), and demonstrates the presence of distinct sets of regulatory factors that may play important roles for gametogenesis in this organism.

Altogether, the stem cell cluster contain ectodermal neuroglandular progenitors (NPC), gastrodermis-derived NPCs (NPC.g), two cell clusters expressing markers indicative of DNA synthesis (mitotic.1) and M phases (mitotic.2) of the mitotic cycle, as well as distinct clusters of early primary germ cells, primary ooctyes and maturing sperm (Fig. 7A and Additional file 2: Fig. S6). NPCs derived from the inner cell layer also express these mitotic markers, further supporting an independent origin of these two cell populations (Fig. 7C, D). The inner cell layer NPCs first appear in the 2d planula samples (Fig. 7B), and are identifiable by *six1/2* expression, previously documented to be enriched in the inner cell layer (Fig. 7D [26]).

### Neuroglandular inventory

**Fig. 8:**
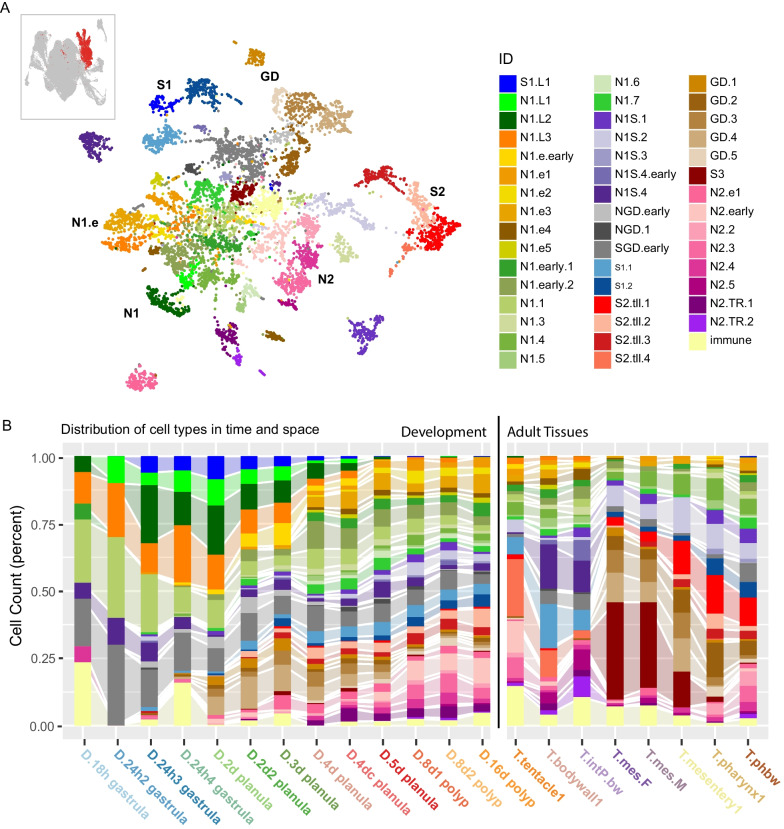
Neuroglandular derivatives include cells of multiple related cell types. **A** Dimensional reduction cell plot (UMAP) showing clustering of the neuroglandular partition (inset). **B** Bar plot of relative contribution of each cell state across all samples

**Fig. 9:**
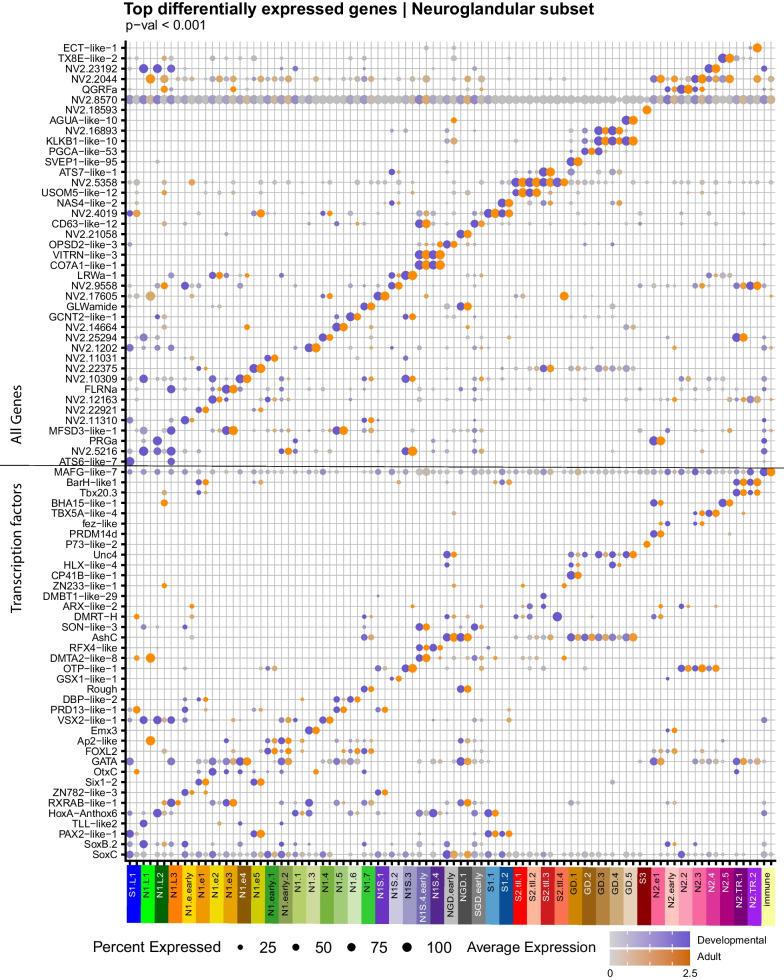
Neuroglandular derivatives include cells of multiple related cell types, continued. Dot plot of gene expression of the top state-specific gene sets from all genes (top) and the set of upregulated transcription factors (bottom). Expression separated between cells of the developmental series (dark slate blue scale) and the adult tissue series (orange scale). Grey indicates average scaled expression of 0 or below. See Additional file 1 for full gene lists

The neural and gland cell types cluster together into a large neuroglandular population, reflective of their close developmental relationships as previously documented. As per [1] here we identify 47 distinct cell states (Fig. 8) which can be broadly separated into *ashC* positive digestive gland cell types (GD: 5 states), *nem64* positive early tentacle retractor cell states (N2.TR: 2 states; [3]), IRF positive putative immune cells (1 state), putative sensory (N1S: 5 states), uncharacterized secretory (S1: 4 states, S2: 4 states, and S3: 1 state), neurons (N: 18 states), and some early progenitor states [7] with elevated *soxB2a* and *soxC* expression (Fig. 9). As described in [1] neurons can be divided into insulinoma (INSM) positive (N1: 16, including 3 larval states (N1.L)) and INSM negative (N2: 6) populations. These can be further sorted according to germ layer origin, where gastrodermis-derived neurons express *six1-2, otx*, and/or *nkx2.2D*. There is only one INSM (–) gastrodermally derived population (N2.g1). All classes of neuroglandular cells reach their peak diversity by the 4d planula. Three larval neural states and one larval S1 state appear at gastrulation and largely disappear once the planula enters the tentacle-bud stage at day 5, although there a few cells in the tentacle and bodywall adult samples that cluster with N1.L3 indicating that this cell type may persist into the adult (Fig. 8B). N1.L1, N1.L2, and S.L1 are instead restricted to the gastrula and planula stages. N1.L2 is likely localized to the oral pole (*six3/6* expression) and may also form part of the apical organ. Gastrodermis-derived neurons appear already in the 2d planula. Only sub-states S2.tll.4 in the tentacles and body wall, and S3 in the mesenteries are restricted to the adult subsets. Early states indicative of tentacle retractor muscle differentiation share expression of *barH* orthologs and *tbx20.3* with inner cell layer neurons N1.g1 (Fig. 9). While the overall composition of the nervous system in *Nematostella* has been described [27–29], to date there are few isolated neural sub-types that have been fully characterized in terms of their molecular fingerprint. Tourniere et al. [30] describe the distribution and close relationship between the *pou4*-expressing N2 neurons and the cnidocytes, and provide evidence of subtypes that we identify here that express RFamide (N2.2), glutamate receptor *GRIK2-like-1* (NVE22966; N2.g1: also described here: [31]), and GABA-A receptor (*GBRB3-like-2*; NVE21438), which here is expressed in the presumed early N2 cell state (N2.early, *SoxB* [2] expressing). Further, the same authors also provide characterization of the N1 subset [32] but do not identity specific neural sub-types. Interestingly they also find an insulinoma-positive (N1 class) subset that is immuno-reactive for RFamide, while here we find this peptide restricted to N2 neurons. However, there are other putative RFamide-peptides in the gene set and so there may be other cross-reactive proteins. We also identify here N1S.4 as the *foxQ2d* expressing sensory neuron described in [33]. Further characterization of this partition is on-going and will likely yield interesting hypotheses regarding the early evolution of neural inventory in the common ancestor of Cnidaria and Bilateria.

### Mucin gland

The mucin-producing cell type is one of the earlier cell types to arise during development, but little is known about its distribution nor function within the organism. This population expresses multiple mucin-like polysaccharides and is one of the first secretory cells to arise in development together with *vsx-*positive early neurons, as previously documented by [1]. Cell states within this cluster appear to be associated with embryo maturation, with 4 states falling within the developmental series and 3 found within the adult. One cell state appears to be restricted to the mesenteries and has a unique transcriptomic profile, including several specific enzymes (eg. *ats10, nas4, loxh1, asprv*: Additional file 1: Figure S8 and Additional file 2: Data 1). This small sub-type also over-expresses RLRa and a number of heat shock proteins (Additional file 1: Figure S8 and Additional file 2: Data 1). Otherwise, the mucin gland cluster is relatively homogeneous and could be considered a single cell type. This cell type is *INSM* positive, reflecting the sister-cell relationship in the hierarchy of specification decisions of the NPCs in the early gastrula [1]. The driver of this cell type identity is likely to be the *noto*-related homeobox gene *not2-like-4*, although this has yet to be proven experimentally. While the gel-forming mucin proteins themselves have been demonstrated to pre-date the cnidarian-bilaterian split [34], analyses of the cell types that produce these proteins is still lacking.

### Immune profile

One novel cell state recovered here is that of the putative immune profile. A large partition of cells exhibits this transcriptomic profile, embedded with the epithelial cloud in reduced dimensional space (Additional file 1: Figure S9). These cells all express elevated levels of *irf1-2a* and *NFKB1* orthologs (Additional file 1: Figure S9). There are 4 stable states associated with this partition, including one cell state that is enriched for histones and other DNA-modulating factors (Additional file 1: Fig. S9, Additional file 2: Supplementary Data 1), but otherwise the transcriptomic signature is largely invariant across the partition. We found cells with this signature also within the neuroglandular partition (Additional file 1: Fig. S9G and Additional file 2: Supplementary Data 1). This transcriptomic state possesses a complex regulatory profile, including a large set of specific transcription factors that includes *nfkb1* (three paralogs), *maf* (three paralogs), and receptors *rlrb* [2 paralogs] and *rlra*. This partition is also enriched in *wnt4* expression, suggesting this wnt as an important modulator of this cell state. Altogether the distribution of this transcriptomic state suggests that it could represent a further distinct cell type that arises from the common neural-glandular progenitor population. Alternatively, this cell state could represent an inducible stress response rather than a distinct cell type. Recent studies have described an inducible immune response with a similar molecular composition (i.e.: IRFs, GBPs, OAS, NFKB Additional file 2: Supplementary Data 1) [35, 36]. Previous investigations into NFKB function in *Nematostella* have shown that protein expression is localized to single cells within the ectoderm of embryos, primary polyps and adults [37, 38], and morpholino knock-down experiments suggest that the NFKB pathway plays a role in cnidocyte development [38]. While we can detect expression of NFKB orthologs within the cnidocyte lineage, it is significantly more abundant within the ectodermal immune partition (Additional file 1: Fig. S9G). However, if this transcriptomic state indeed reflects a previously undescribed immune cell type in sea anemone's that similarly arise from the common neuroglandular stem cell population, perturbations could potentially affect multiple cell derivatives, including the cnidocytes. Brennan et al. [39] have demonstrated that the NFKB pathway is also active in the nematosomes, ball-like assemblies of stinging cells that are released into the body cavity of *Nematostella*, presumably from the septal filaments. Cells with an amebocyte-like morphology that are capable of phagocytosis have also been isolated from *Nematostella* [40]. Of particular interest, we find a second cell state associated with the gastrodermis which expresses the paralogs *irf1-2b* and *irf1-2c* but otherwise show very little overlap with the ectodermal immune state (Additional file 1: Fig. S9G). Similarly in corals two transcriptomic states with molecular signatures indicative of immune function, including *irf1/2* expression, have also been cataloged within the single cell transcriptomic atlas [41]. In corals the immune cell states also express multiple secreted proteins, whereas here the more abundant ectodermal immune profile in the anemone does not. The gastrodermal immune state however does express elevated levels of cytoskeletal proteins and peptidases (Additional file 2: Supplementary Data1). Further experimental investigation of these cell states is warranted to distinguish between these two alternatives: an inducible cell state(s) vs. dedicated immune cell type(s). Nonetheless, it is clear from the cell atlas data presented here that two robust immune-related cell states are present in *Nematostella*, and these are distinct within both germ layers.

## Conclusions

Here we provide an updated wildtype single cell atlas of development and tissue composition for the starlet sea anemone *Nematostella vectensis*. We identify a total of 127 distinct transcriptomic states within the dataset, comprising both unique cell types as well as developmental cell states. This is similar to the first single cell transcriptomic atlas produced for this species, wherein 104 unique transcriptomic states were described from adult tissues and 39 from a larval stage, of which 23 overlapped with the adult inventory [2]. The neuroglandular partition is transcriptomically by far the most diverse, here accounting for 37% of the cell states, while comprising only 9% of the entire dataset. We can correlate transcriptomic states of the inner cell layer with anatomical structures (different muscle groups, membrane attachment of the mesenteries, inner lining of the pharynx), and similarly the ectodermal layer can be separated into the inner ectodermal or pharyngeal layer and the outer epidermis. However, we find few transcriptomic states reflective of distinct cell types associated with the large epithelial partitions, but rather separations that are reflective of maturation of these tissues from the embryonic state (18–48 h) into the adult state that is maintained after embryogenesis has completed. While we can fully recapitulate the annotations provide in our previous works [1, 3], we extend our observations of the data to include further characterization of the putative stem cell population, including providing a full series of sperm maturation that has yet to be fully analysed, and the identification of two separate immune-like profiles associated with both germ layers. We hope that the updated catalog of transcriptomic states provided here will serve the community in its quest to understand not only *Nematostella* biology, but also as a valuable resource for use in comparative analyses of cell type composition across evolutionary time.

## Methods

### Additional data

Cell suspensions were generated according to [1] for an additional 24 h gastrula sample (~ 100 animals), an additional 2d planula (~ 50 animals), and an additional mesentery sample that was harvested from an adult male after a regular spawning cycle. Nonetheless, the mesentery was washed multiple times to reduce the amount of sperm present in the capture. All samples were first washed in Ca/Mg-free artificial sea water [42]. The 24h and mesentery samples were dissociated in equal volumes of 3.75 mg/ml Papain and 1.25 mg/ml collagenase in PBS for one hour without agitation, followed by mechanical disruption by pipetting briefly every 15 min for an additional hour. Successful single cell suspensions were monitored under a compound microscope. Digestion was stopped by dilution with 1 ml of PBS, and cells were collected via centrifugation at 400 RCF for 10 min. 2d planula samples were processed with 10X TrypLE Select according to [1]. Cell pellets after washing were re-suspended in PBS with 0.5% BSA, diluted to a concentration of 1000 cells/uL after assessment using ViaStainTM AOPI Staining Solution (Nexcelom, #CS2-0106) to measure viability with a Cellometer X2 (Nexcelom). All samples had a viability of at least 80%. Raw data from these cell captures have been deposited into the GEO repository (GSE200198).

### Mapping

Raw sequence data from all samples (GSE200198 and GSE154105) were mapped to the Nv2 gene models and accompanying vs.2 chromosome genome assembly ([4] https://simrbase.stowers.org/jb_pub/?data=data/starlet_pub) using the 10 × genomics cell ranger pipeline, without secondary analysis and force recovery of 10,000 cells per library. Gene nomenclature was updated with the following priority: gene names found in the literature, best-blast hit from automated gene annotations (“-like-integer”), gene model. A look up table for conversion between gene names and gene models (the latest Nv2, and the former NVE) is provided in Supplemental Data 2:genes.LUT.

### Single cell analyses

The UMI-reduced gene by cell matrices were then imported into R and filtered for cells containing at least 250 UMIs. Putative multiplets were identified as outliers at the upper end of the UMI content and removed for each sample prior to concatenating all data matrices together by gene rows. Analysis and clustering strategy follows that reported in [1], and the R script can be accessed on our GitHub (https://github.com/technau/Nv2_Atlas). Briefly, expression matrices from each sample were concatenated, the data was then log normalized using a scale factor of 5000 (Seurat::NormalizeData). The gene set containing the top 1000 variable features from each sample was identified (5188 genes total), and all genes of the set were scaled within each sample separately (Seurat::ScaleData, with ‘split.by’ parameter). The scaled data slot was then used as input into the Seurat::RunPCA function, and finally 30 dimensions were used as input for both Seurat::FindNeighbors and Seurat::RunUMAP. The entire dataset was then clustered into 12 major cell-type partitions (resolution = 0.2) and each partition was then re-analyzed separately to identify transcriptomic profiles (clusters) with robust unique gene sets. For each subset the top 2000 variable gene set was identified (Seurat::FindVariableFeatures), these were scaled and centered by sample across the full dataset (Seurat::ScaleData, split.by = 'orig.ident') and copied into the scale.data slot of the subset. For each subset 20 principal components were selected as input for the neighbourhood graph and UMAP dimension reduction. The neuroglandular subset was the exception, where 3000 variable genes were identified, and 30 components were retained. Clustering was then performed with a resolution value of 0.2 and a semi-automated cluster name assignment was performed with the marker gene sets found in Supplemental Data [2], as described in [1]. Briefly, upregulated marker genes were collated and assigned a nomenclature (cell state ID). This collated gene list was then used to identify which of the genes showed the highest average expression, and the corresponding cell state ID was then assigned to that cluster. Separate analyses of the tissue layers were similarly performed on data subsets containing multiple partitions. The epithelial subset includes partitions identified as ‘embryonic ectoderm’, ‘pharyngeal ectoderm’ and ‘epithelial ectoderm’. From this subset, one cluster was identified as corresponding to the apical organ of the planula larvae based on public gene expression profiles. This subset was also isolated and analyzed separately as above, but with only 10 principle components retained. The gastroderm subset includes partitions identified as ‘embryonic mesendoderm’ and ‘gastroderm’. UMAP reduction cell plots with underlying expression matrix will be available for exploration on the UCSC Cellbrowser website (https://sea-anemone-atlas.cells.ucsc.edu).

### Supplementary Information

**Additional file 1. Supplementary Figures S1:** S9 Complementary data figures for all partitions not further illustrated in the main manuscript. A) Partition identity highlighted on UMAP of the full dataset B) UMAP cell plot coloured by sample identity C) Barplot of absolute cell numbers in each sample, coloured by cell state identity. D) UMAP cell plot coloured by cell state identity. E, F) Dotplot expression of top five marker genes (E) and differentially expressed transcription factors (F) from each cluster. Expression separated between cells of the developmental series (Dark slate blue scale) and the adult tissue series (orange scale). Grey indicates average scaled expression of 0 or below. See Supplementary material for full gene lists. S9G Dotplot expression profile of specific immune related regulatory genes across the entire dataset. The signature is found within the immune partition (orange) but also in the immune-cells of the neuroglandular partition (box in green partition), but not shared with the putative immune signature of the inner cell layer (box in pink partition).

**Additional file 2. Supplementary Data 1:** Differentially expressed gene lists from set of all genes and only putative transcription factors for all clusters described in the paper. Worksheet 1 includes the cell-state ID for all barcodes in the dataset.

**Additional file 3. Supplementary Data 2:** Marker genes used for semi-automated name assignments for each cluster subset described in the paper. Worksheet 1 includes a look up table that includes the new gene model, the nomenclature used here, and the previous gene model(s).

## Data Availability

The datasets generated and analysed during the current study are available in the Gene expression Omnibus (GEO) repository [GSE200198 and GSE154105], https://www.ncbi.nlm.nih.gov/geo
