# Supplementary material for: Updated single cell reference atlas for the starlet anemone Nematostella vectensis
Source: Front Zool. 2024 Mar 18;21:8. doi: 10.1186/s12983-024-00529-z (PMC10946136; doi:10.1186/s12983-024-00529-z)
Supplement: Supplementary file 1 — Additional file 1. Supplementary Figures S1: S9 Complementary data figures for all partitions not further illustrated in the main manuscript. A) Partition identity highlighted on UMAP of the full dataset B) UMAP cell plot coloured by sample identity C) Barplot of absolute cell numbers in each sample, coloured by cell state identity. D) UMAP cell plot coloured by cell state identity. E, F) Dotplot expression of top five marker genes (E) and differentially expressed transcription factors (F) from each cluster. Expression separated between cells of the developmental series (Dark slate blue scale) and the adult tissue series (orange scale). Grey indicates average scaled expression of 0 or below. See Supplementary material for full gene lists. S9G Dotplot expression profile of specific immune related regulatory genes across the entire dataset. The signature is found within the immune partition (orange) but also in the immune-cells of the neuroglandular partition (box in green partition), but not shared with the putative immune signature of the inner cell layer (box in pink partition). [file 12983_2024_529_MOESM1_ESM.pdf]

Updated single cell reference atlas for the starlet anemone *Nematostella vectensis*.

Alison G. Cole[1,2], Julia Steger[1], Julia Hagauer[1], Andreas Denner[1], Patricio Ferrer Murguia[1], Paul Knabl[1], Sanjay Narayanaswamy[1], Brittney Wick[3], Juan D. Montenegro[1], Ulrich Technau[1,2,4]

Affiliations:

[1] Department of Neurosciences and Developmental Biology, Faculty of Life Sciences, University of Vienna, Djerassiplatz 1, 1030 Vienna.

[2] Research platform Single Cell Regulation of Stem Cells, University of Vienna, Djerassiplatz 1, 1030 Vienna.

[3] UCSC Cellbrowser, University of California, Santa Cruz, USA.

[4] Max Perutz labs, University of Vienna, Dr. Bohrgasse 9, 1090 Vienna

Corresponding authors: alison.cole@univie.ac.at and ulrich.technau@univie.ac.at

Supplementary Figures S1:S9

Complementary data figures for all partitions not further illustrated in the main manuscript. **A)** Partition identity highlighted on UMAP of the full dataset **B)** UMAP cell plot coloured by sample identity **C)** Barplot of absolute cell numbers in each sample, coloured by cell state identity. **D)** UMAP cell plot coloured by cell state identity. **E,F**) Dotplot expression of top five marker genes (E) and differentially expressed transcription factors (F) from each cluster. Expression separated between cells of the developmental series (Dark slate blue scale) and the adult tissue series (orange scale). Grey indicates average scaled expression of 0 or below. See Supplementary material for full gene lists. **S9G** Dotplot expression profile of specific immune related regulatory genes across the entire dataset. The signature is found within the immune partition (orange) but also in the immune-cells of the neuroglandular partition (box in green partition), but not shared with the putative immune signature of the inner cell layer (box in pink partition).

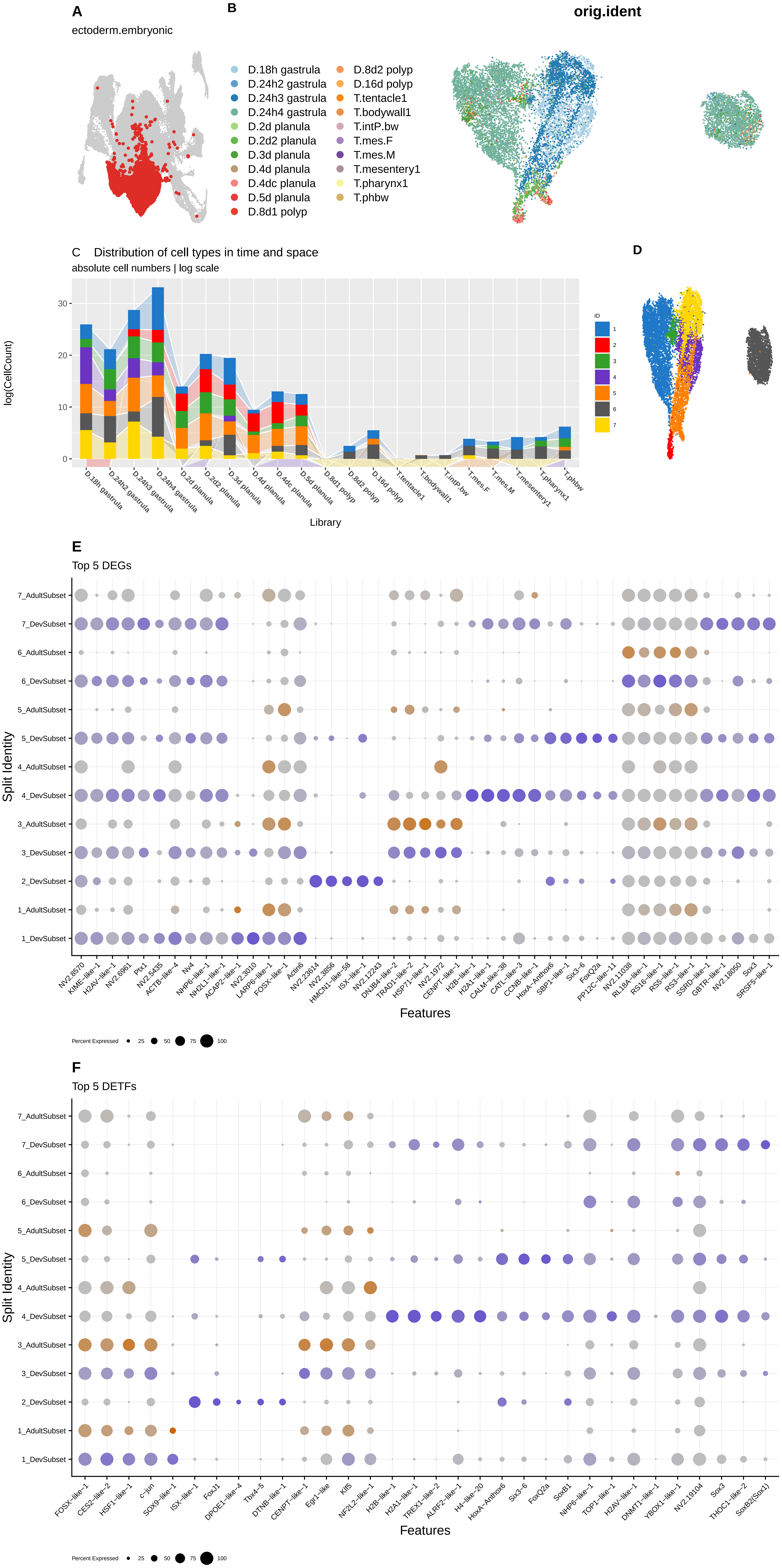
A
ectoderm.embryonic
B
orig.ident
D.18h gastrula
D.24h2 gastrula
D.24h3 gastrula
D.24h4 gastrula
D.2d planula
D.2d2 planula
D.3d planula
D.4d planula
D.4dc planula
D.5d planula
D.8d1 polyp
D.8d2 polyp
D.16d polyp
T.tentacle1
T.bodywall1
T.intP.bw
T.mes.F
T.mes.M
T.mesentery1
T.pharynx1
T.phbw
C Distribution of cell types in time and space
absolute cell numbers | log scale
log(CellCount)
Library
D
ID
E
Top 5 DEGs
Split Identity
Features
Percent Expressed 25 50 75 100
F
Top 5 DETFs
Split Identity
Features
Percent Expressed 25 50 75 100

Figure S1

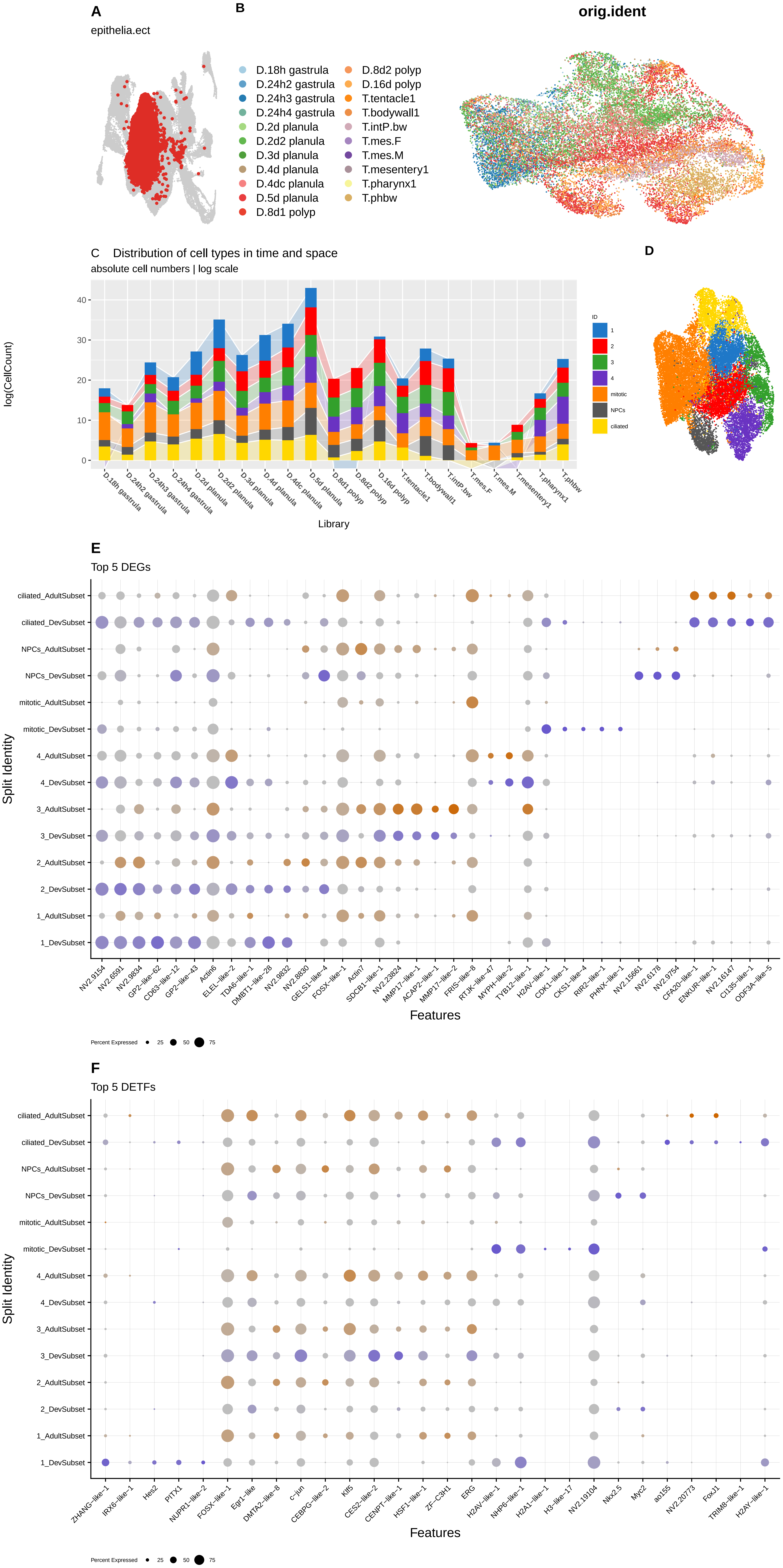
A
epithelia.ect
B
orig.ident
D.18h gastrula
D.24h2 gastrula
D.24h3 gastrula
D.24h4 gastrula
D.2d planula
D.2d2 planula
D.3d planula
D.4d planula
D.4dc planula
D.5d planula
D.8d1 polyp
D.8d2 polyp
D.16d polyp
T.tentacle1
T.bodywall1
T.intP.bw
T.mes.F
T.mes.M
T.mesentery1
T.pharynx1
T.phbw
C Distribution of cell types in time and space
absolute cell numbers | log scale
log(CellCount)
Library
ID
1
2
3
4
mitotic
NPCs
ciliated
D
E
Top 5 DEGs
Split Identity
Features
Percent Expressed
25
50
75
F
Top 5 DETFs
Split Identity
Features
Percent Expressed
25
50
75

Figure S2

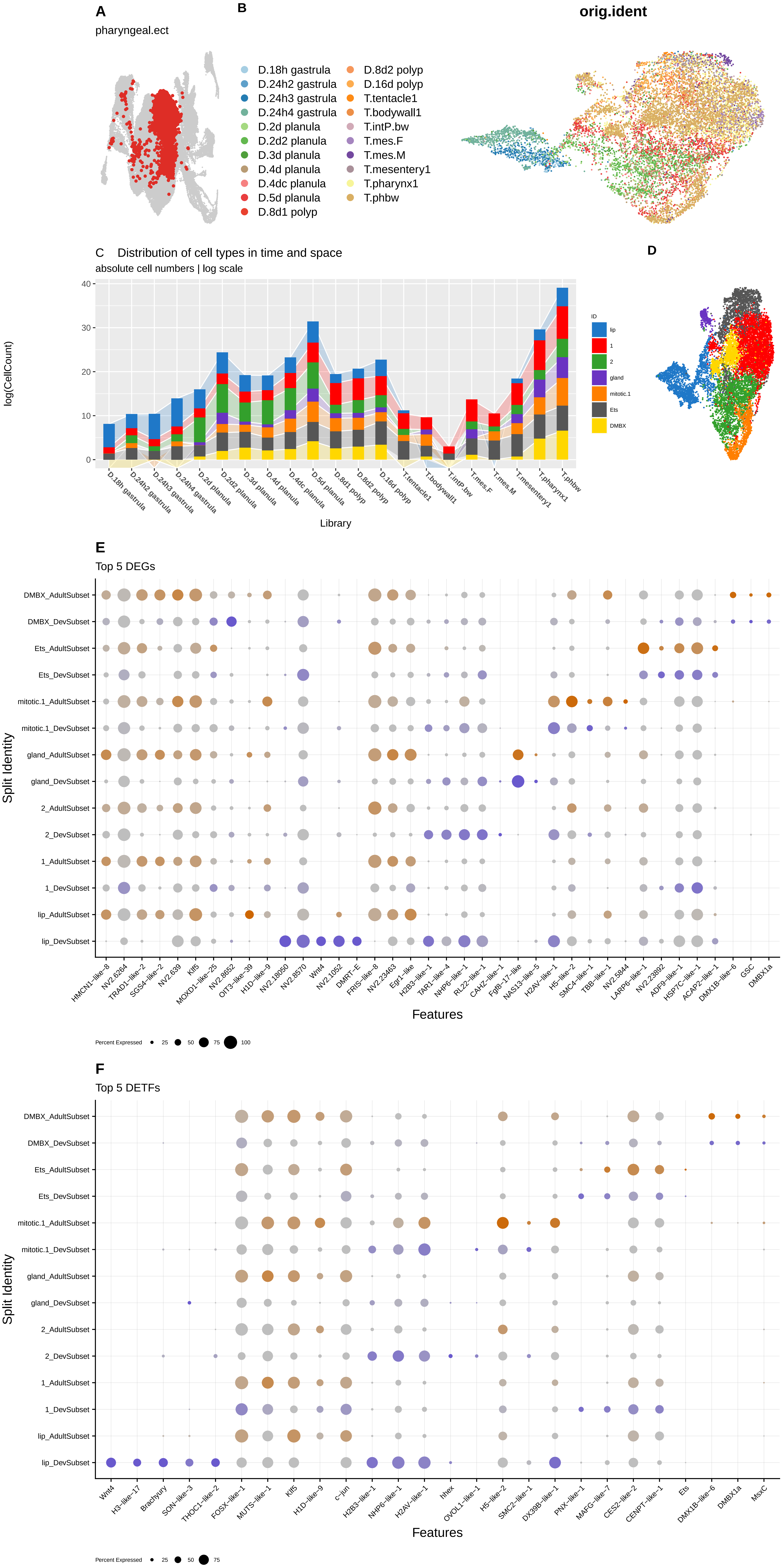
A
pharyngeal.ect
B
orig.ident
D.18h gastrula
D.24h2 gastrula
D.24h3 gastrula
D.24h4 gastrula
D.2d planula
D.2d2 planula
D.3d planula
D.4d planula
D.4dc planula
D.5d planula
D.8d1 polyp
D.8d2 polyp
D.16d polyp
T.tentacle1
T.bodywall1
T.intP.bw
T.mes.F
T.mes.M
T.mesentery1
T.pharynx1
T.phbw
C Distribution of cell types in time and space
absolute cell numbers | log scale
log(CellCount)
Library
ID
lip
1
2
gland
mitotic.1
Ets
DMBX
D
E
Top 5 DEGs
Split Identity
DMBX_AdultSubset
DMBX_DevSubset
Ets_AdultSubset
Ets_DevSubset
mitotic.1_AdultSubset
mitotic.1_DevSubset
gland_AdultSubset
gland_DevSubset
2_AdultSubset
2_DevSubset
1_AdultSubset
1_DevSubset
lip_AdultSubset
lip_DevSubset
HMCN1−like−8
NV2.6264
TRAD1−like−2
SGS4−like−2
NV2.639
Klf5
MOXD1−like−25
NV2.8652
OIT3−like−39
H1D−like−9
NV2.18050
NV2.8570
Wnt4
NV2.1052
DMRT−E
FRIS−like−8
NV2.23463
Egr1−like
H2B3−like−1
TAR1−like−4
NHP6−like−1
RL22−like−1
CAHZ−like−1
Fgf8−17−like
NAS13−like−5
H2AV−like−1
H5−like−2
SMC4−like−1
TBB−like−1
NV2.5844
LARP6−like−1
NV2.23892
ADF9−like−1
HSP7C−like−1
ACAP2−like−1
DMX1B−like−6
GSC
DMBX1a
Features
Percent Expressed
25
50
75
100
F
Top 5 DETFs
Split Identity
Wnt4
H3−like−17
Brachyury
SON−like−3
THOC1−like−2
FOSX−like−1
MUTS−like−1
Klf5
H1D−like−9
c−jun
H2B3−like−1
NHP6−like−1
H2AV−like−1
hhex
OVOL1−like−1
H5−like−2
SMC2−like−1
DX39B−like−1
PNX−like−1
MAFG−like−7
CES2−like−2
CENPT−like−1
Ets
DMX1B−like−6
DMBX1a
MsxC
Features
Percent Expressed
25
50
75

Figure S3

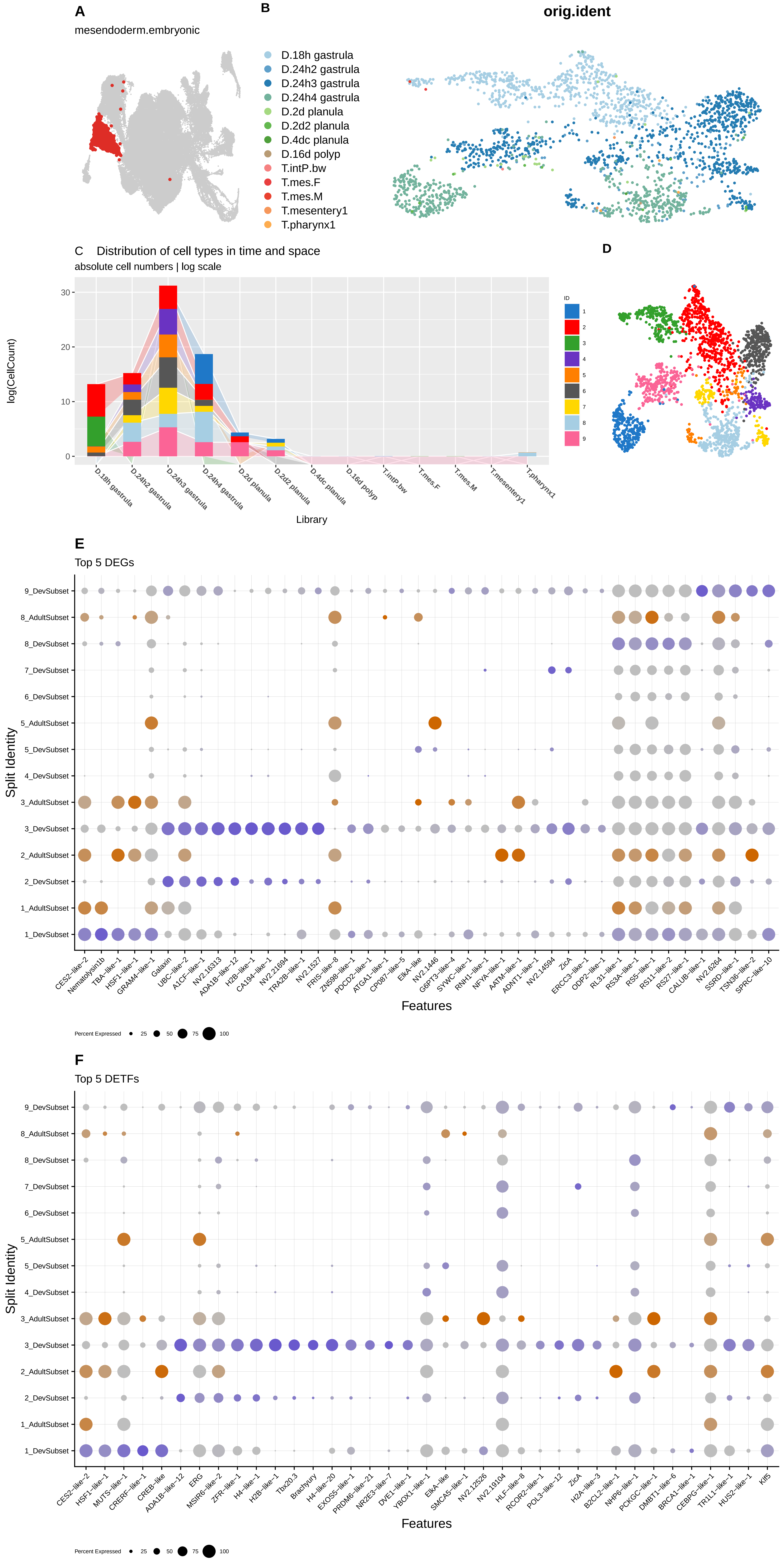
A
mesendoderm.embryonic
B
orig.ident
D.18h gastrula
D.24h2 gastrula
D.24h3 gastrula
D.24h4 gastrula
D.2d planula
D.2d2 planula
D.4dc planula
D.16d polyp
T.intP.bw
T.mes.F
T.mes.M
T.mesentery1
T.pharynx1
C Distribution of cell types in time and space
absolute cell numbers | log scale
log(CellCount)
Library
D
ID
E
Top 5 DEGs
Split Identity
9_DevSubset
8_AdultSubset
8_DevSubset
7_DevSubset
6_DevSubset
5_AdultSubset
5_DevSubset
4_DevSubset
3_AdultSubset
3_DevSubset
2_AdultSubset
2_DevSubset
1_AdultSubset
1_DevSubset
Features
CES2−like−2
Nematolysin1b
TBA−like−1
HSF1−like−1
GRAM4−like−1
Galaxin
UBC−like−2
A1CF−like−1
NV2.16313
ADA1B−like−12
H2B−like−1
CA194−like−1
NV2.21694
TRA2B−like−1
NV2.1527
FRIS−like−8
ZN598−like−1
PDCD2−like−1
ATGA1−like−1
CP087−like−5
ElkA−like
NV2.1446
G6PT3−like−4
SYWC−like−1
RNH1−like−1
NFYA−like−1
AATM−like−1
ADNT1−like−1
NV2.14594
ZicA
ERCC3−like−1
ODP2−like−1
RL31−like−1
RS3A−like−1
RS5−like−1
RS11−like−2
RS27−like−1
CALUB−like−1
NV2.6264
SSRD−like−1
TSN36−like−2
SPRC−like−10
Percent Expressed 25 50 75 100
F
Top 5 DETFs
CES2−like−1
HSF1−like−1
MUTS−like−1
CRERF−like−1
CREB−like
ADA1B−like−12
ERG
MSIR6−like−2
ZFR−like−1
H4−like−1
H2B−like−1
Tbx20.3
Brachyury
H4−like−20
EXOS5−like−1
PRDM6−like−21
NR2E3−like−7
DVE1−like−1
YBOX1−like−1
ElkA−like
SMCA5−like−1
NV2.12526
NV2.19104
HLF−like−8
RCOR2−like−1
POL3−like−12
ZicA
H2A−like−3
B2CL2−like−1
NHP6−like−1
PCKGC−like−1
DMBT1−like−6
BRCA1−like−1
CEBPG−like−1
TR1L1−like−1
HUS2−like−1
Klf5

Figure S4

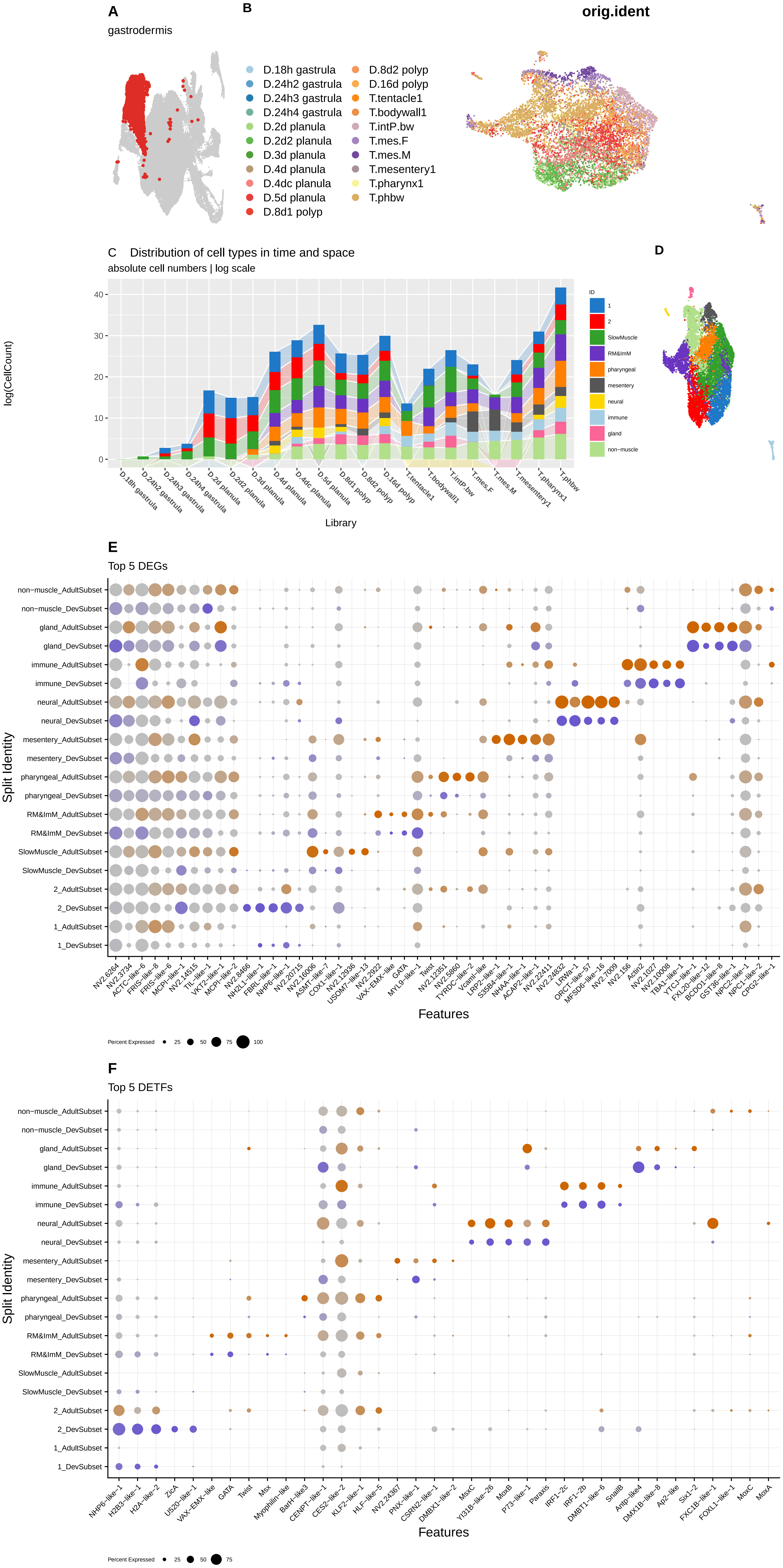

A
gastrodermis
B
orig.ident
D.18h gastrula
D.24h2 gastrula
D.24h3 gastrula
D.24h4 gastrula
D.2d planula
D.2d2 planula
D.3d planula
D.4d planula
D.4dc planula
D.5d planula
D.8d1 polyp
D.8d2 polyp
D.16d polyp
T.tentacle1
T.bodywall1
T.intP.bw
T.mes.F
T.mes.M
T.mesentery1
T.pharynx1
T.phbw
C Distribution of cell types in time and space
absolute cell numbers | log scale
log(CellCount)
Library
D
ID
1
2
SlowMuscle
RM&ImM
pharyngeal
mesentery
neural
immune
gland
non−muscle
E
Top 5 DEGs
Split Identity
non−muscle_AdultSubset
non−muscle_DevSubset
gland_AdultSubset
gland_DevSubset
immune_AdultSubset
immune_DevSubset
neural_AdultSubset
neural_DevSubset
mesentery_AdultSubset
mesentery_DevSubset
pharyngeal_AdultSubset
pharyngeal_DevSubset
RM&ImM_AdultSubset
RM&ImM_DevSubset
SlowMuscle_AdultSubset
SlowMuscle_DevSubset
2_AdultSubset
2_DevSubset
1_AdultSubset
1_DevSubset
Features
Percent Expressed 25 50 75 100
F
Top 5 DETFs
Split Identity
Features
Percent Expressed 25 50 75

Figure S5

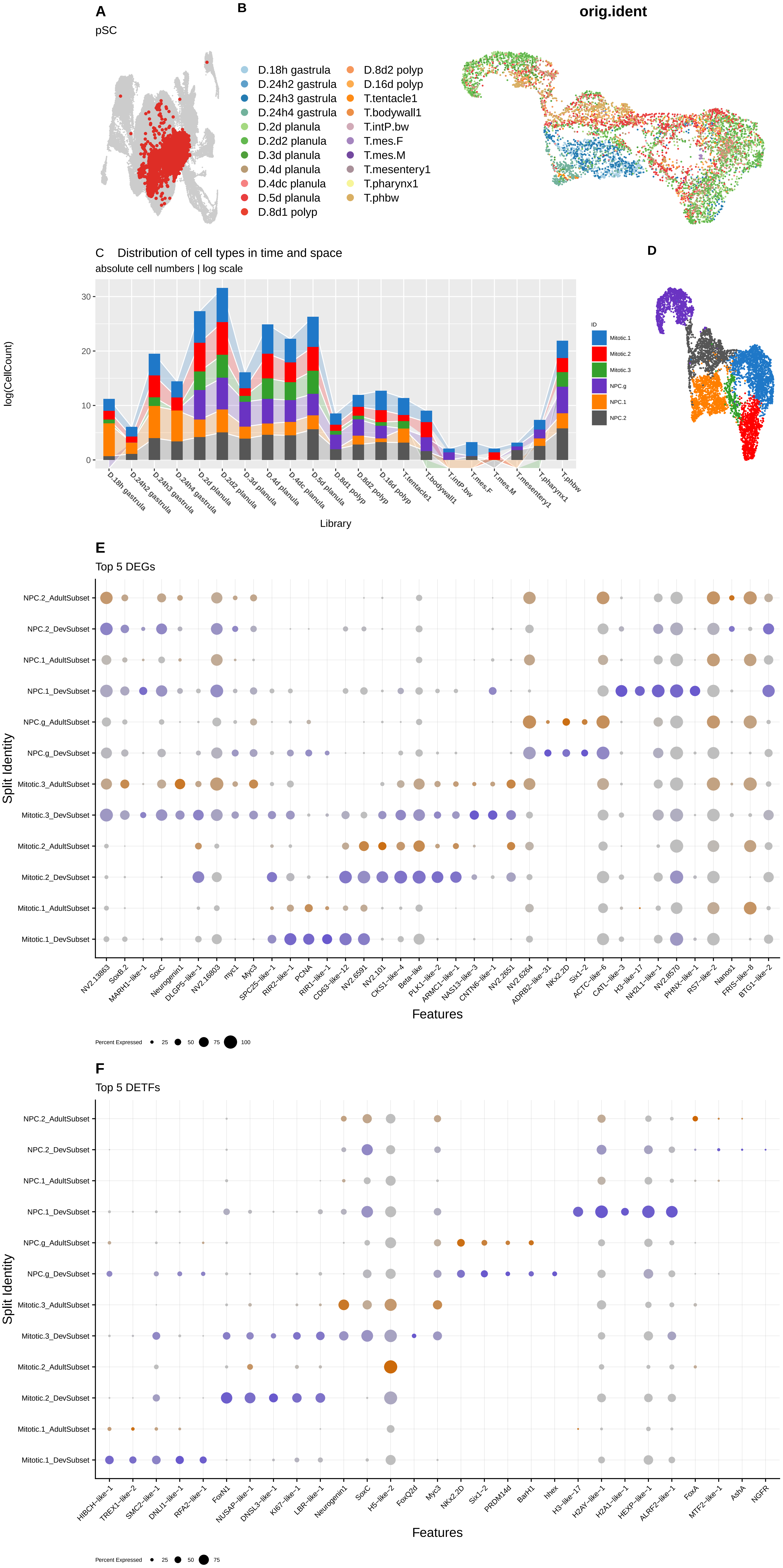
A
pSC
B
orig.ident
D.18h gastrula
D.24h2 gastrula
D.24h3 gastrula
D.24h4 gastrula
D.2d planula
D.2d2 planula
D.3d planula
D.4d planula
D.4dc planula
D.5d planula
D.8d1 polyp
D.8d2 polyp
D.16d polyp
T.tentacle1
T.bodywall1
T.intP.bw
T.mes.F
T.mes.M
T.mesentery1
T.pharynx1
T.phbw
C Distribution of cell types in time and space
absolute cell numbers | log scale
log(CellCount)
Library
ID
Mitotic.1
Mitotic.2
Mitotic.3
NPC.g
NPC.1
NPC.2
D
E
Top 5 DEGs
Split Identity
NPC.2_AdultSubset
NPC.2_DevSubset
NPC.1_AdultSubset
NPC.1_DevSubset
NPC.g_AdultSubset
NPC.g_DevSubset
Mitotic.3_AdultSubset
Mitotic.3_DevSubset
Mitotic.2_AdultSubset
Mitotic.2_DevSubset
Mitotic.1_AdultSubset
Mitotic.1_DevSubset
NV2.13863
SoxB.2
MARH1−like−1
SoxC
Neurogenin1
DLGP5−like−1
NV2.16803
myc1
Myc3
SPC25−like−1
RIR2−like−1
PCNA
RIR1−like−1
CD63−like−12
NV2.6591
NV2.101
CKS1−like−4
Beta−like
PLK1−like−2
ARMC1−like−1
NAS13−like−3
CNTN6−like−1
NV2.2651
NV2.6264
ADRB2−like−31
NKx2.2D
Six1−2
ACTC−like−6
CATL−like−3
H3−like−17
NH2L1−like−1
NV2.8570
PHNX−like−1
RS7−like−2
Nanos1
FRIS−like−8
BTG1−like−2
Features
Percent Expressed
F
Top 5 DETFs
HIBCH−like−1
TREX1−like−2
SMC2−like−1
DNLI1−like−1
RFA2−like−1
FoxN1
NUSAP−like−1
DNSL3−like−1
KI67−like−1
LBR−like−1
Neurogenin1
SoxC
H5−like−2
FoxQ2d
Myc3
NKx2.2D
Six1−2
PRDM14d
BarH1
hhex
H3−like−17
H2AY−like−1
H2A1−like−1
HEXP−like−1
ALRF2−like−1
FoxA
MTF2−like−1
AshA
NGFR

Figure S6

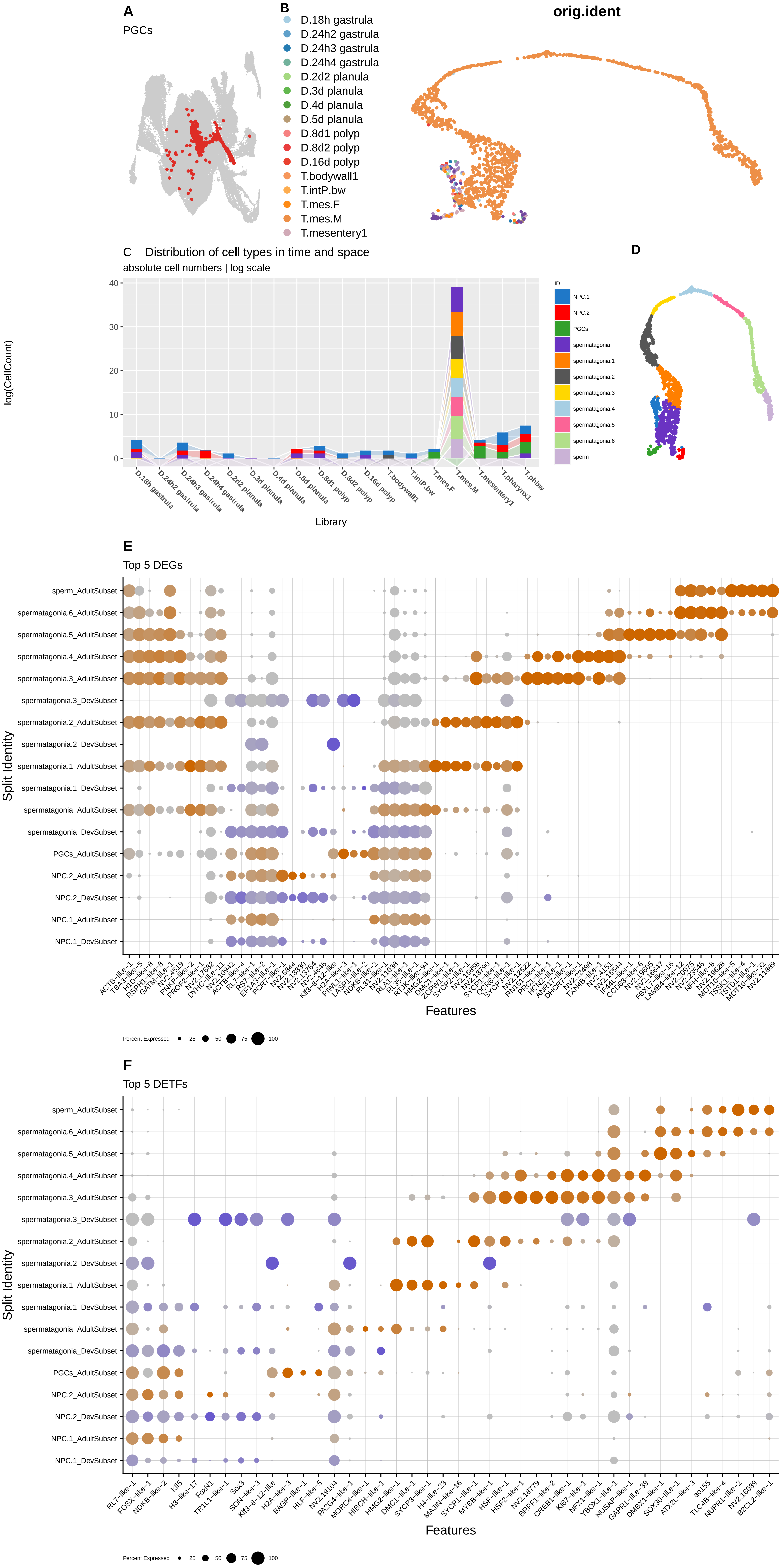

A
PGCs
B
orig.ident
D.18h gastrula
D.24h2 gastrula
D.24h3 gastrula
D.24h4 gastrula
D.2d2 planula
D.3d planula
D.4d planula
D.5d planula
D.8d1 polyp
D.8d2 polyp
D.16d polyp
T.bodywall1
T.intP.bw
T.mes.F
T.mes.M
T.mesentery1
C Distribution of cell types in time and space
absolute cell numbers | log scale
log(CellCount)
Library
D.18h gastrula
D.24h2 gastrula
D.24h3 gastrula
D.24h4 gastrula
D.2d2 planula
D.3d planula
D.4d planula
D.5d planula
D.8d1 polyp
D.8d2 polyp
D.16d polyp
T.bodywall1
T.intP.bw
T.mes.F
T.mes.M
T.mesentery1
T.pharynx1
T.phbw
ID
NPC.1
NPC.2
PGCs
spermatagonia
spermatagonia.1
spermatagonia.2
spermatagonia.3
spermatagonia.4
spermatagonia.5
spermatagonia.6
sperm
D
E
Top 5 DEGs
Split Identity
Features
sperm_AdultSubset
spermatagonia.6_AdultSubset
spermatagonia.5_AdultSubset
spermatagonia.4_AdultSubset
spermatagonia.3_AdultSubset
spermatagonia.3_DevSubset
spermatagonia.2_AdultSubset
spermatagonia.2_DevSubset
spermatagonia.1_AdultSubset
spermatagonia.1_DevSubset
spermatagonia_AdultSubset
spermatagonia_DevSubset
PGCs_AdultSubset
NPC.2_AdultSubset
NPC.2_DevSubset
NPC.1_AdultSubset
NPC.1_DevSubset
Percent Expressed 25 50 75 100
F
Top 5 DETFs
Split Identity
Features
Percent Expressed 25 50 75 100

Figure S7

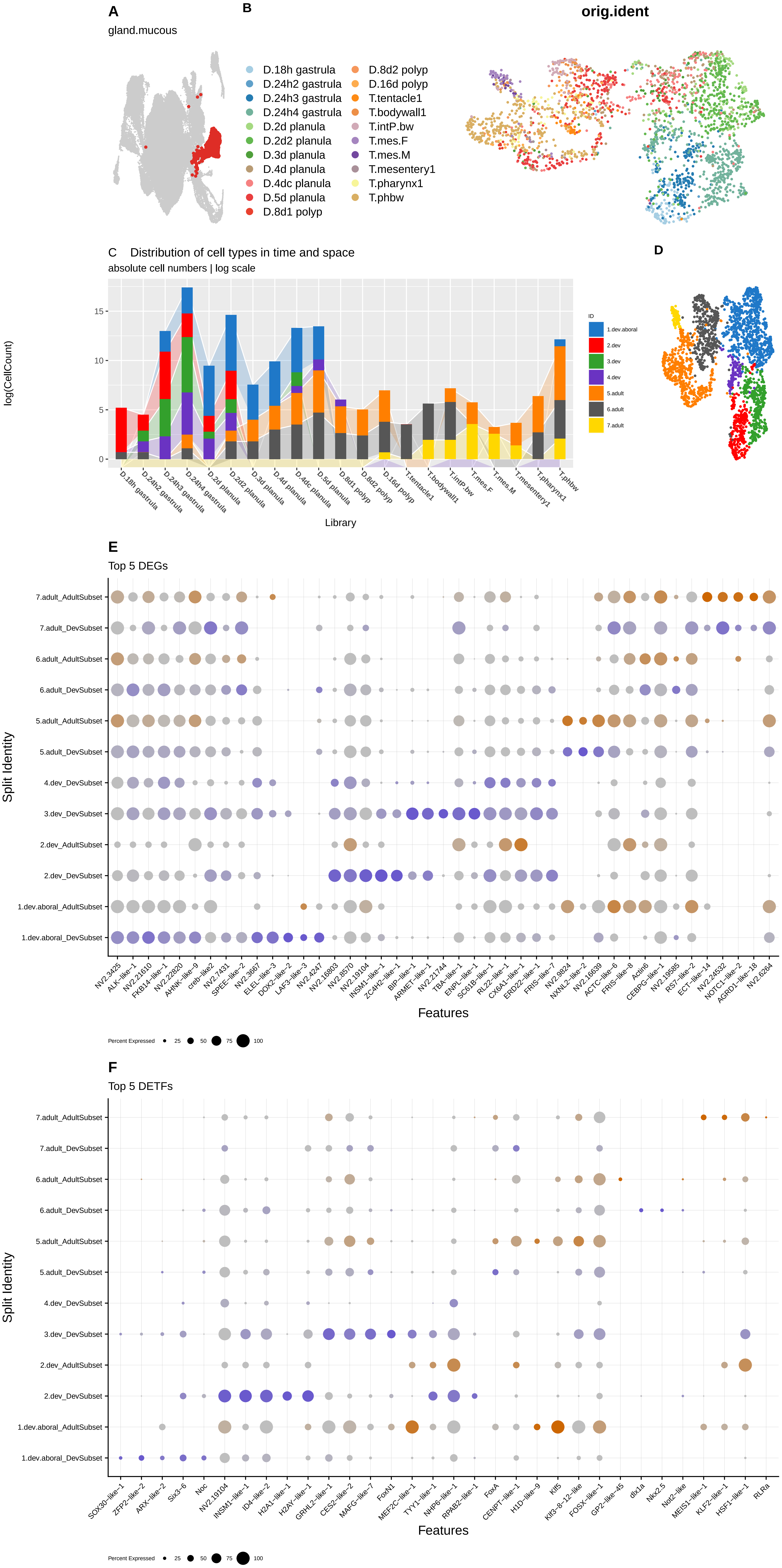

A
gland.mucous
B
orig.ident
D.18h gastrula
D.24h2 gastrula
D.24h3 gastrula
D.24h4 gastrula
D.2d planula
D.2d2 planula
D.3d planula
D.4d planula
D.4dc planula
D.5d planula
D.8d1 polyp
D.8d2 polyp
D.16d polyp
T.tentacle1
T.bodywall1
T.intP.bw
T.mes.F
T.mes.M
T.mesentery1
T.pharynx1
T.phbw
C Distribution of cell types in time and space
absolute cell numbers | log scale
log(CellCount)
Library
D
ID
1.dev.aboral
2.dev
3.dev
4.dev
5.adult
6.adult
7.adult
E
Top 5 DEGs
Split Identity
7.adult_AdultSubset
7.adult_DevSubset
6.adult_AdultSubset
6.adult_DevSubset
5.adult_AdultSubset
5.adult_DevSubset
4.dev_DevSubset
3.dev_DevSubset
2.dev_AdultSubset
2.dev_DevSubset
1.dev.aboral_AdultSubset
1.dev.aboral_DevSubset
Features
Percent Expressed 25 50 75 100
F
Top 5 DETFs
Split Identity
Features
Percent Expressed 25 50 75 100

Figure S8

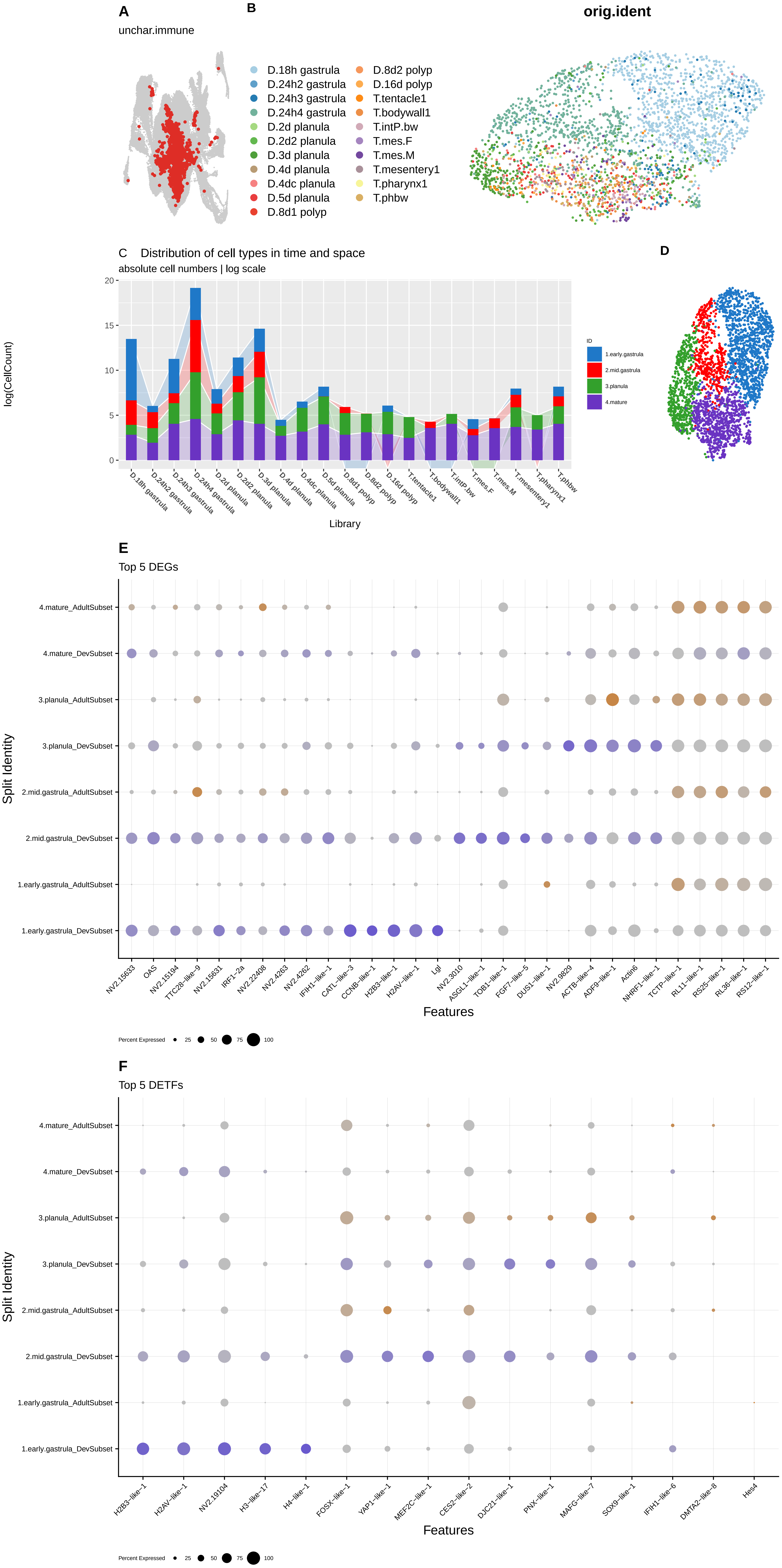
A
unchar.immune
B
orig.ident
D.18h gastrula
D.24h2 gastrula
D.24h3 gastrula
D.24h4 gastrula
D.2d planula
D.2d2 planula
D.3d planula
D.4d planula
D.4dc planula
D.5d planula
D.8d1 polyp
D.8d2 polyp
D.16d polyp
T.tentacle1
T.bodywall1
T.intP.bw
T.mes.F
T.mes.M
T.mesentery1
T.pharynx1
T.phbw
C Distribution of cell types in time and space
absolute cell numbers | log scale
log(CellCount)
Library
ID
1.early.gastrula
2.mid.gastrula
3.planula
4.mature
D
E
Top 5 DEGs
Split Identity
4.mature_AdultSubset
4.mature_DevSubset
3.planula_AdultSubset
3.planula_DevSubset
2.mid.gastrula_AdultSubset
2.mid.gastrula_DevSubset
1.early.gastrula_AdultSubset
1.early.gastrula_DevSubset
NV2.15633
OAS
NV2.15194
TTC28−like−9
NV2.15631
IRF1−2a
NV2.22408
NV2.4263
NV2.4262
IFIH1−like−1
CATL−like−3
CCNB−like−1
H2B3−like−1
H2AV−like−1
Lgl
NV2.3010
ASGL1−like−1
TOB1−like−1
FGF7−like−5
DUS1−like−1
NV2.9829
ACTB−like−4
ADF9−like−1
Actin6
NHRF1−like−1
TCTP−like−1
RL11−like−1
RS25−like−1
RL36−like−1
RS12−like−1
Features
Percent Expressed 25 50 75 100
F
Top 5 DETFs
Split Identity
H2B3−like−1
H2AV−like−1
NV2.19104
H3−like−17
H4−like−1
FOSX−like−1
YAP1−like−1
MEF2C−like−1
CES2−like−2
DJC21−like−1
PNX−like−1
MAFG−like−7
SOX9−like−1
IFIH1−like−6
DMTA2−like−8
Hes4
Features
Percent Expressed 25 50 75 100

Figure S9

G Ectodermal immune signature is found as a cell state within the neuroglandular complement. The Gastrodermal immune signature is distinct

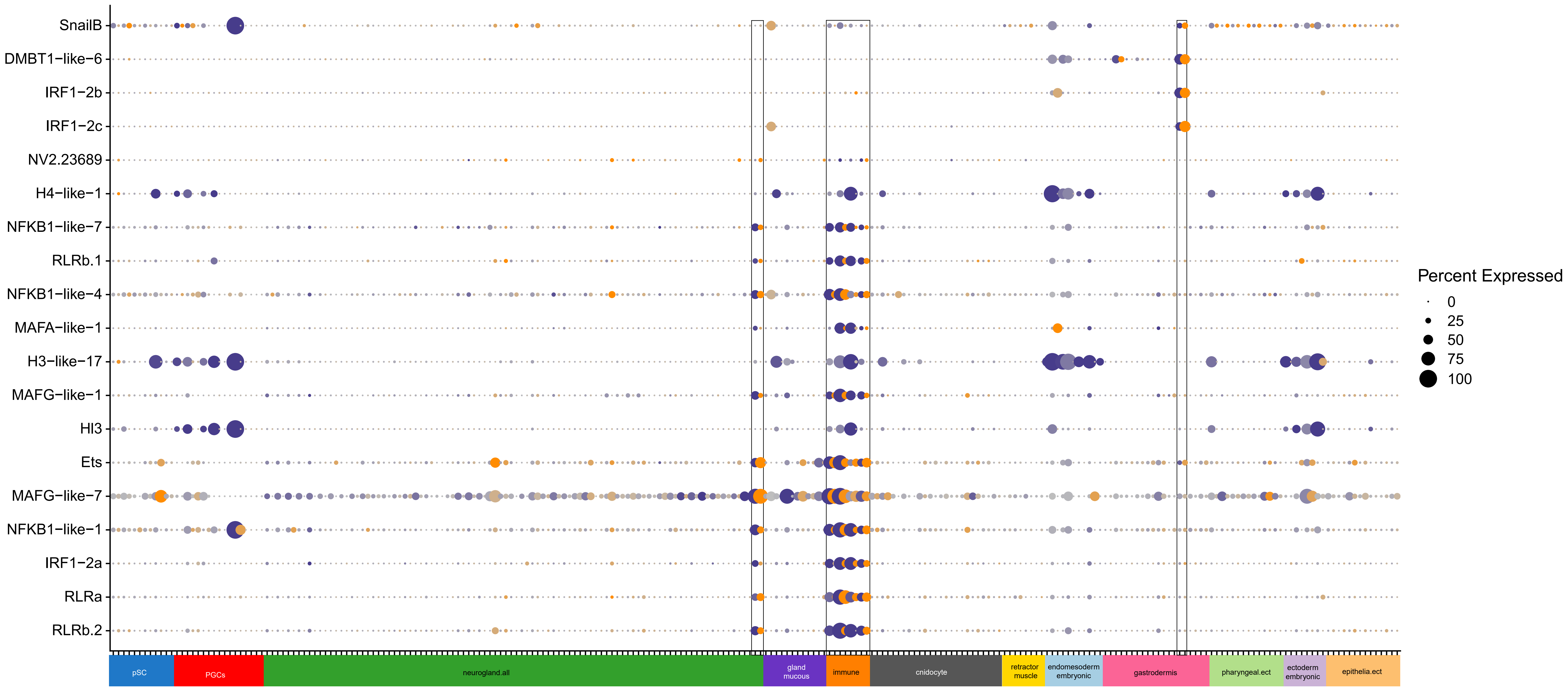

Supplementary Figures S1:9

Complementary data figures for all partitions not further illustrated in the main manuscript. A) Partition identity highlighted on UMAP of the full dataset B) UMAP cell plot coloured by sample identity C) Barplot of absolute cell numbers in each sample, coloured by cell state identity. D) UMAP cell plot coloured by cell state identity. E,F) Dotplot expression of top five marker genes (E) and differentially expressed transcription factors (F) from each cluster. Expression separated between cells of the developmental series (Dark slate blue scale) and the adult tissue series (orange scale). Grey indicates average scaled expression of 0 or below. See Supplementary material for full gene lists. S9G Dotplot expression profile of specific immune related regulatory genes across the entire dataset. The signature is found within the immune partition (orange) but also in the immune-cells of the neuroglandular partition (box in green partition), but not shared with the putative immune signature of the inner cell layer (box in pink partition).

Figure S9 con't
